# The effect of hospital teaching status on adverse outcomes among breast cancer patients receiving radiotherapy and brachytherapy

**DOI:** 10.1097/MD.0000000000044648

**Published:** 2025-10-03

**Authors:** Yimei Zhong, Bingxin Liu, Jifang Zhou, Yali Ge, Na Rui, Liliang Shen, Fan Yang

**Affiliations:** a School of International Business, China Pharmaceutical University, Nanjing, Jiangsu, China; b Department of Anesthesiology, Perioperative and Pain Medicine, Nanjing First Hospital, Nanjing Medical University, Nanjing, Jiangsu, China; c Department of Education, Nanjing First Hospital, Nanjing Medical University, Nanjing, Jiangsu, China; d The Affiliated People’s Hospital of Ningbo University, Zhejiang, China; e School of Science, China Pharmaceutical University, Nanjing, Jiangsu, China.

**Keywords:** brachytherapy, breast cancer, hospital teaching status, Nationwide Readmissions Database, radiotherapy

## Abstract

Radiotherapy and brachytherapy are integral treatments for breast cancer, yet the influence of hospital characteristics on patient outcomes remains underexplored. This study evaluated the influence of hospital teaching status (teaching vs non-teaching) on radiation-related complications, in-hospital mortality, readmission rate and healthcare resource utilization (HCRU) in breast cancer patients receiving these therapies. We conducted a retrospective cohort study using the US Nationwide Readmissions Database (2016–2021), including female breast cancer patients aged ≥18 years who underwent radiotherapy or brachytherapy. Multivariable logistic regression models evaluated the impact of hospital teaching status (teaching vs non-teaching) on clinical outcomes. Among 9030 patients, 7177 (79.5%) were treated at teaching hospitals. These patients were younger, had a higher prevalence of metastatic disease, and were less likely to undergo breast-conserving surgery or mastectomy. In-hospital mortality was higher for radiotherapy than for brachytherapy (4.2% vs 0.3%), with no unadjusted difference by hospital type. Radiation-related complications were more frequent in non-teaching hospitals for both radiotherapy (OR: 1.11 [1.01, 1.21]). Radiotherapy readmission rates were lower at non-teaching hospitals at 30 (OR: 0.82 [0.70, 0.95]) and 90 days (OR: 0.84 [0.74, 0.95]), with reduced costs and shorter length of stay compared to patients receiving radiotherapy at teaching hospitals. Our study demonstrates that breast cancer patients undergoing radiotherapy at non-teaching hospitals experienced better prognosis, with lower readmission rates, reduced healthcare costs, and shorter lengths of stay compared to those treated at teaching hospitals. These findings highlight important differences in patient outcomes and healthcare resource utilization between teaching and non-teaching hospitals, emphasizing the need for further research to understand the underlying causes and optimize care delivery.

## 1. Introduction

Breast cancer remains a significant public health concern worldwide, particularly among women,^[1]^ due to its prevalence and potential for severe health implications. It is the most common malignancy affecting women and a leading cause of morbidity, disability and mortality in women worldwide.^[2]^ It has already surpassed lung cancer as the most commonly diagnosed cancer, with an estimated 2.3 million new cases (11.7%) in 2020.^[3]^

Given the high incidence and severe consequences of breast cancer, effective treatment strategies are critical. In recent years, brachytherapy has gained traction as an alternative for breast cancer.^[4,5]^ Breast brachytherapy is the placement of radioactive sources inside breast tissue to deliver a relatively high dose of radiation either to gross tumor or to the volume of tissue surrounding the site where the tumor originated and from which it was excised.^[6]^ Every year, numerous patients around the world benefit from brachytherapy.^[7]^ Compared with radiotherapy, brachytherapy course is typically several weeks shorter, thereby reducing inconvenience associated with radiation treatment,^[8]^ and may potentially decrease toxicity to distant breast tissue.^[4]^ Despite its efficacy and safety as a non-pharmacological treatment has been demonstrated,^[7]^ the short-term radiation-related complications and readmission rates associated with brachytherapy are not well characterized.

Hospital selection plays a crucial role in cancer patient outcomes, receiving complex cancer treatments at under-resourced hospitals may increase the risk of adverse outcomes.^[9,10]^ Understanding the relationship between hospital characteristics and the prognosis of breast cancer patients undergoing radiotherapy and brachytherapy could provide valuable insights for reducing adverse treatment outcomes. However, few studies to date have evaluated the impact of hospital characteristics on the prognosis and healthcare resource utilization of breast cancer patients treated with radiotherapy and brachytherapy.

Therefore, this study aimed to investigate the relationship between hospital characteristics, particularly hospital teaching status (teaching vs non-teaching type), and the prevalence of radiation-related complications, in-hospital mortality, readmission outcomes, and healthcare resource utilization among breast cancer patients undergoing radiotherapy and brachytherapy. The findings of this study were expected to provide critical insights into reducing adverse outcomes in breast cancer patients treated with radiotherapy and brachytherapy, while also offering valuable guidance for optimizing healthcare resource utilization and reducing medical costs.

## 2. Methods

### 2.1. Data sources

We conducted a retrospective cohort study using the 2016 to 2021 Nationwide Readmissions Databases (NRD). The NRD is created using the State Inpatient Databases from geographically dispersed states, representing approximately 50% of the total US resident population and 50% of all US hospitalizations. It includes all discharge records of patients treated in US community hospitals, excluding rehabilitation and long-term acute care facilities. Discharge weights are provided to obtain national estimates, with unweighted data comprising about 15 million discharges per year and weighted data estimating roughly 35 million discharges annually. The NRD contains verified patient linkage numbers that track individuals across hospitals within a state while protecting privacy, though these numbers do not allow tracking of the same person across different years.

### 2.2. Study population

**Figure 1.**
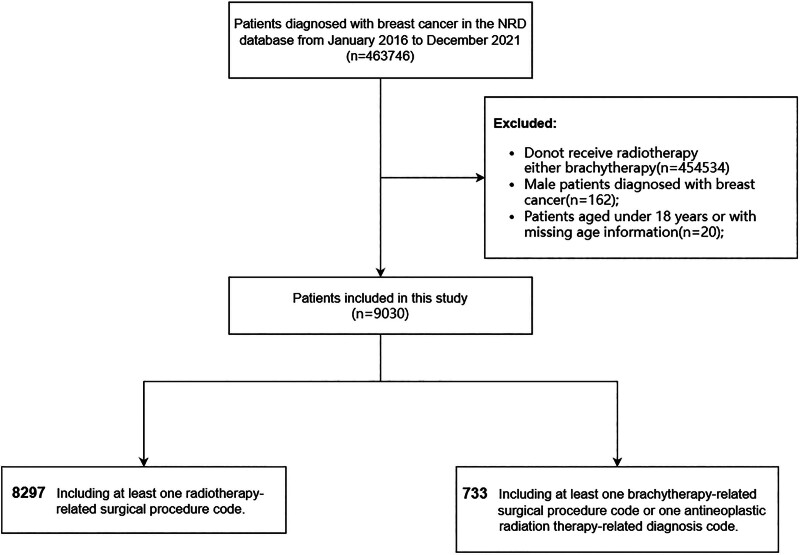
Study flow diagram.

Patients diagnosed with BC who underwent radiotherapy or brachytherapy between January 1, 2016, and December 31, 2021, based on international classification of diseases, tenth revision, clinical modification (ICD-10-CM) codes. Radiotherapy and brachytherapy were distinguished using procedure codes, supplemented by diagnostic codes (Y84.2, Z51.0, W88.1XXA; see Supplemental_Brachytherapy_Radiotherapy_ICD10). Patients younger than 18 years or male were excluded. The patient selection process is detailed in Figure 1. To ensure precise cohort stratification, we relied on procedure codes. Initially, we planned to categorize patients into 3 groups: radiotherapy-only, brachytherapy only, and radiotherapy or brachytherapy. However, as diagnostic codes could not reliably distinguish radiotherapy from brachytherapy, and the brachytherapy-only group was too small for separate analysis, we ultimately defined 2 cohorts: a radiotherapy cohort (based on radiotherapy procedure codes) and a brachytherapy cohort (brachytherapy ± radiotherapy, based on brachytherapy procedure codes, including patients with potential concurrent radiotherapy diagnostic codes).

### 2.3. Exposure

The exposure variable of the study was hospital teaching status (teaching vs non-teaching type). Teaching hospitals are institutions affiliated with medical schools and serve as training sites for residents and medical students, which is identified by the “HOSP_UR_TEACH” data element in the HCUP database.

### 2.4. Study outcomes

The primary outcomes of interest were radiation-related complications, in-hospital mortality, and all-cause readmission rates. In-hospital mortality was defined as death from any cause occurring after the index date, regardless of whether it was directly related to breast cancer. All-cause readmission rates were defined as the proportion of patients who were readmission for any reason within 30- or 90-days following discharge after the index date, as recorded in the NRD. Secondary outcomes included length of stay (LOS) and total hospital costs. Radiation-related complications include radiation-induced wounds, infections, strokes, thrombotic events, gastrointestinal issues, cardiovascular and respiratory diseases, neurological disorders, skin conditions, pulmonary diseases, lymphedema, shoulder joint disorders, or unspecified conditions, patients with any of these were classified as having a radiation-related complication.

### 2.5. Covariate definitions

Baseline covariates included patient- and hospital-level characteristics. Patient characteristics included demographics (age, year, LOS, and insurance type and income), comorbidities (chronic obstructive pulmonary disease (COPD), depression, diabetes, hypertension, hypothyroidism, obesity, anemic, coagulopathy, neutropenia, nausea and vomiting, metabolic disorders, AKI, CKD, coronary artery disease, atrial fibrillation (AF), liver disease, pneumonia, congestive, endocarditis, personal history of irradiation, smoking, alcohol use and Charlson Comorbidity Index score). Comorbidities were ascertained using the ICD-10-CM (Table S1, Supplemental Digital Content, https://links.lww.com/MD/Q51). Hospital characteristics included hospital location, bed size, and ownership.

### 2.6. Statistical analysis

Differences in patient-level and hospital-level characteristics between teaching and non-teaching hospitals were assessed in our analysis. For categorical variables, we employed Pearson chi-square test and Fisher exact test. Pearson chi-square test was used for variables with large sample sizes and expected frequencies of ≥5 to evaluate significant associations between variables. Fisher exact test was applied when sample sizes were small or when expected frequencies were <5 to ensure the accuracy and reliability of the results.

Student *t* test compared means between 2 independent groups for continuous variables, while the Mann–Whitney *U* test was used for non-normal data. Categorical data are reported as frequencies and percentages, while continuous data are presented as means with standard deviations (SD) or medians with interquartile ranges (IQR).

Multivariable logistic regression models were used to evaluate the association between clinical outcomes and hospital teaching status. Results were reported as odds ratios with 95% confidence intervals (CIs). To further optimize the model fit, we computed and compared the Akaike Information Criterion (AIC) values across 5 models (Models 1–5) to evaluate the goodness-of-fit of each model. A lower AIC value indicates better model fit. The results of this comparison are presented in Table S12, Supplemental Digital Content, https://links.lww.com/MD/Q51.

Additionally, to refine the selection of variables for the model, we performed stepwise regression analysis based on AIC for the fully adjusted model. This analysis aimed to identify the optimal set of covariates to enhance the model’s predictive accuracy. The results of the stepwise regression, including the selected variables, AIC values, and *P*-values for hospital teaching status, are provided in Table S13, Supplemental Digital Content, https://links.lww.com/MD/Q51.

Additionally, quantile regression models were used to assess the impact of hospital teaching status on additional costs and LOS. Quantile regression is a robust method for estimating conditional quantiles, such as the median, and is advantageous over traditional linear regression as it does not rely on assumptions of normality and homoscedasticity. This method is particularly useful for analyzing skewed or non-normally distributed data, such as hospital costs and LOS. We applied patient-specific weights to calculate weighted expenditures and LOS. In all models, teaching hospitals were designated as the reference group for comparison.

All statistical analyses were performed using SAS version 9.2 (Cary, NC: SAS Institute, Inc.) and R version 4.3.3 (R Foundation for Statistical Computing, Vienna, Austria). A two-sided significance level of α < 0.05 was considered statistically significant.

## 3. Results

### 3.1. Treatment trends

This study included 9030 female breast cancer patients treated with either brachytherapy or radiotherapy from January 1, 2016, to September 30, 2021 (Fig. 1). Of these, 7177 (79.5%) were treated at teaching hospitals and 1853 (20.5%) at non-teaching hospitals, with radiotherapy patients comprising 91.7% and 92.5% of these groups, respectively.

**Figure 2.**
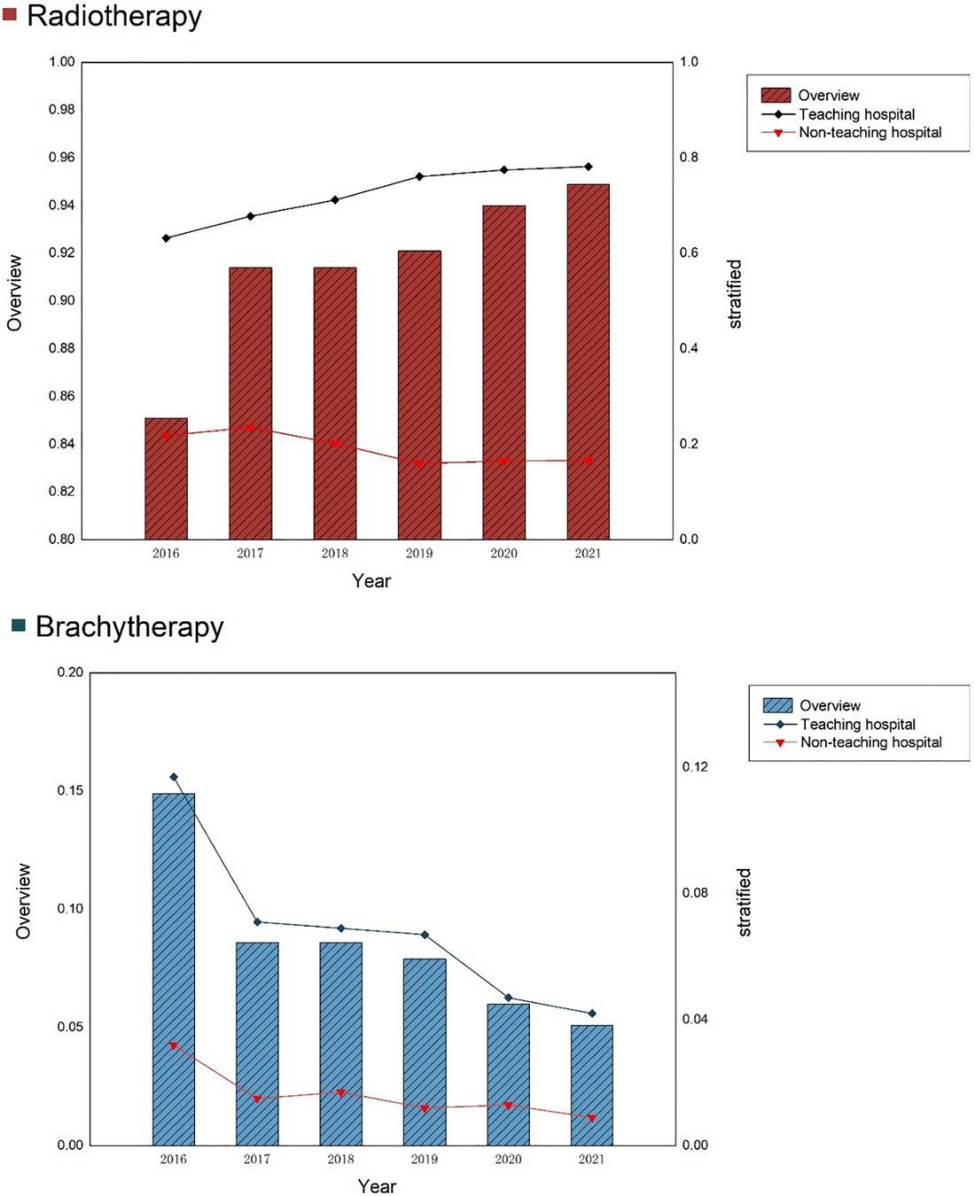
Temporal trends in the proportion of treatment modalities stratified by hospital teaching status from 2016 to 2021.

The proportion of breast cancer patients receiving radiotherapy consistently increased, particularly in teaching hospitals, where it rose from 63.2% in 2016 to 78.2% in 2021. Conversely, non-teaching hospitals experienced an overall decline. Meanwhile, the proportion of patients receiving brachytherapy decreased overall, dropping from 11.7% in 2016 to 4.2% in 2021 in teaching hospitals, with similar declines observed in non-teaching hospitals (as shown in Table 1 and Fig. 2).

**Table 1:** Temporal trends in the proportion of treatment modalities stratified by hospital teaching status from 2016 to 2021.

	2016 (N = 925)	2017 (N = 1700)	2018 (N = 1657)	2019 (N = 1771)	2020 (N = 1492)	2021 (N = 1485)
	n (%)	n (%)	n (%)	n (%)	n (%)	n (%)
Overall						
Teaching hospital	693 (74.9)	1273 (74.9)	1294 (78.1)	1466 (82.8)	1226 (82.2)	1225 (82.5)
Non-teaching hospital	232 (25.1)	427 (25.1)	363 (21.9)	305 (17.2)	266 (17.8)	260(17.5)
Radiotherapy-only						
Teaching and non-teaching hospital	787 (85.1)	1554 (91.4)	1514 (91.4)	1631 (92.1)	1402 (94.0)	1409 (94.9)
Teaching hospital	585 (63.2)	1153 (67.8)	1179 (71.2)	1348 (76.1)	1156 (77.5)	1162 (78.2)
Non-teaching hospital	202 (21.8)	401 (23.6)	335 (20.2)	283 (16.0)	246 (16.5)	247 (16.6)
Brachytherapy ± radiotherapy						
Teaching & non-teaching hospital	138 (14.9)	146 (8.6)	143 (8.6)	140 (7.9)	90 (6.0)	76 (5.1)
Teaching hospital	108 (11.7)	120 (7.1)	115 (6.9)	118 (6.7)	70 (4.7)	63 (4.2)
Non-teaching hospital	30 (3.2)	26 (1.5)	28 (1.7)	22 (1.2)	20 (1.3)	13 (0.9)

### 3.2. Baseline characteristics

The baseline characteristics of breast cancer patients receiving radiotherapy and brachytherapy were compared between teaching and non-teaching hospitals (Table 2).

**Table 2:** Baseline characteristics of BC patients stratified by treatment types and hospital teaching status.

	Overall			Radiotherapy-only			Brachytherapy ± Radiotherapy		
	Teaching hospital (N = 7177)	Non-teaching hospital (N = 1853)	*P*-value	Teaching hospital (N = 6583)	Non-teaching hospital (N = 1714)	*P*-value	Teaching hospital (N = 594)	Non-teaching hospital (N = 139)	*P*-value
	n (%)	n (%)		n (%)	n (%)		n (%)	n (%)	
Age (yr), median (IQR)	61 (51, 70)	64 (54, 73)	<.001	61 (51, 70)	64 (54, 73)	<.001	59 (49, 67)	62 (51, 71)	.015
Age at admission									
18–24 yr	7 (0.1)	0 (0.0)	<.001	5 (0.1)	0 (0.0)	<.001	2 (0.3)	0 (0.0)	.034
25–34 yr	187 (2.6)	28 (1.5)		177 (2.7)	26 (1.5)		10 (1.7)	2 (1.4)	
35–44 yr	785 (10.9)	126 (6.8)		702 (10.7)	111 (6.5)		83 (14.0)	15 (10.8)	
45–54 yr	1455 (20.3)	329 (17.8)		1323 (20.1)	305 (17.8)		132 (22.2)	24 (17.3)	
55–64 yr	1957 (27.3)	459 (24.8)		1784 (27.1)	419 (24.4)		173 (29.1)	40 (28.8)	
65–74 yr	1694 (23.6)	523 (28.2)		1564 (23.8)	483 (28.2)		130 (21.9)	40 (28.8)	
75+ yr	1092 (15.2)	388 (20.9)		1028 (15.6)	370 (21.6)		64 (10.8)	18 (12.9)	
Year									
2016	693 (9.7)	232 (12.5)	<.001	585 (8.9)	202 (11.8)	<.001	108 (18.2)	30 (21.6)	.580
2017	1273 (17.7)	427 (23.0)		1153 (17.5)	401 (23.4)		120 (20.2)	26 (18.7)	
2018	1294 (18.0)	363 (19.6)		1179 (17.9)	335 (19.5)		115 (19.4)	28 (20.1)	
2019	1466 (20.4)	305 (16.5)		1348 (20.5)	283 (16.5)		118 (19.9)	22 (15.8)	
2020	1226 (17.1)	266 (14.4)		1156 (17.6)	246 (14.4)		70 (11.8)	20 (14.4)	
2021	1225 (17.1)	260 (14.0)		1162 (17.7)	247 (14.4)		63 (10.6)	13 (9.4)	
LOS, mean (SD)	6.6 (10.00)	5.5 (7.23)	<.001	7.0 (10.3)	5.7 (7.4)	<.001	2.3 (4.5)	2.1 (2.2)	.572
Cost, mean (SD)	26,466.3 (36,854.3)	21,191.5 (22,240.3)	<.001	26,817.0 (38,047.9)	21,065.4 (22,553.1)	<.001	22,591.7 (18,835.9)	22,755.4 (17,906.5)	.926
Insurance type									
Medicare	3027 (42.2)	977 (52.8)	<.001	2830 (43.0)	917 (53.5)	<.001	197 (33.2)	60 (43.2)	.047
Medicaid	1134 (15.8)	211 (11.4)		1058 (16.1)	198 (11.6)		76 (12.8)	13 (9.4)	
Private insurance	2781 (38.8)	620 (33.5)		2481 (37.7)	556 (32.5)		300 (50.5)	64 (46.0)	
Self-pay	76 (1.1)	7 (0.4)		72 (1.1)	7 (0.4)		4 (0.7)	0 (0.0)	
No charge	8 (0.1)	3 (0.2)		7 (0.1)	3 (0.2)		1 (0.2)	0 (0.0)	
Other	142 (2.0)	34 (1.8)		126 (1.9)	32 (1.9)		16 (2.7)	2 (1.4)	
Income quartile*									
1st quartile (lowest)	1484 (20.9)	425 (23.3)	<.001	1384 (21.3)	401 (23.7)	<.001	100 (17.0)	24 (17.6)	.360
2st quartile	1601 (22.6)	506 (27.7)		1443 (22.2)	466 (27.6)		158 (26.9)	40 (29.4)	
3st quartile	1827 (25.8)	437 (23.9)		1688 (26.0)	402 (23.8)		139 (23.6)	35 (25.7)	
4st quartile (highest)	2178 (30.7)	457 (25.0)		1987 (30.6)	420 (24.9)		191 (32.5)	37 (27.2)	
Clinical characteristics									
Metastatic cancer	3556 (49.5)	820 (44.3)	<.001	3433 (52.1)	793 (46.3)	<.001	123 (20.7)	27 (19.4)	.736
Breast conservatory surgery†	411 (5.7)	143 (7.7)	.002	323 (4.9)	121 (7.1)	.001	88 (14.8)	22 (15.8)	.764
Mastectomy‡	1753 (24.4)	480 (25.9)	.199	1314 (20.0)	382 (22.3)	.036	439 (73.9)	98 (70.5)	.415
Chemotherapy§	183 (2.5)	10 (0.5)	<.001	182 (2.8)	10 (0.6)	<.001	1 (0.2)	0 (0.0)	.629
CCI									
0–4	1503 (22.6)	353 (20.1)	.002	1245 (20.5)	305 (18.9)	.011	258 (43.6)	48 (34.8)	.256
5–6	1075 (16.1)	342 (19.5)		934 (15.4)	299 (18.5)		141 (23.8)	43 (31.2)	
7–8	759 (11.4)	216 (12.3)		692 (11.4)	196 (12.1)		67 (11.3)	20 (14.5)	
9+	3321 (49.9)	844 (48.1)		3195 (52.7)	817 (50.5)		126 (21.3)	27 (19.6)	
Comorbidities									
COPD	181 (2.5)	60 (3.2)	.104	180 (2.7)	60 (3.5)	.108	1 (0.2)	0 (0.0)	.629
Depression	143 (2.0)	47 (2.5)	.173	134 (2.0)	45 (2.6)	.160	9 (1.5)	2 (1.4)	.947
Diabetes	777 (10.8)	193 (10.4)	.641	712 (10.8)	176 (10.3)	.542	65 (10.9)	17 (12.2)	.665
Hypertension	357 (5.0)	105 (5.7)	.252	344 (5.2)	99 (5.8)	.400	13 (2.2)	6 (4.3)	.156
Hypothyroidism	1004 (14.0)	292 (15.8)	.058	952 (14.5)	278 (16.2)	.074	52 (8.8)	14 (10.1)	.625
Alcohol abuse	60 (0.8)	19 (1.0)	.522	59 (0.9)	19 (1.1)	.502	1 (0.2)	0 (0.0)	.629
Smoker	2034 (28.3)	506 (27.3)	.394	1883 (28.6)	472 (27.5)	.400	151 (25.4)	34 (24.5)	.815
Obesity	722 (10.1)	142 (7.7)	.002	657 (10.0)	134 (7.8)	.008	65 (10.9)	8 (5.8)	.066
Anemic-deficiency	1049 (14.6)	296 (16.0)	.154	1017 (15.4)	287 (16.7)	.202	32 (5.4)	9 (6.5)	.616
Coagulopathy	280 (3.9)	72 (3.9)	1.000	275 (4.2)	71 (4.1)	1.000	5 (0.8)	1 (0.7)	.886
Neutropenia	315 (4.4)	69 (3.7)	.230	314 (4.8)	68 (4.0)	.178	1 (0.2)	1 (0.7)	.263
Nausea and vomiting	371 (5.2)	86 (4.6)	.387	346 (5.3)	84 (4.9)	.596	25 (4.2)	2 (1.4)	.119
Metabolic disorders	100 (1.4)	30 (1.6)	.537	100 (1.5)	30 (1.8)	.564	0 (0.0)	0 (0.0)	NA
AKI	679 (9.5)	160 (8.6)	.295	668 (10.1)	158 (9.2)	.272	11 (1.9)	2 (1.4)	.740
CKD	287 (4.0)	73 (3.9)	.960	274 (4.2)	72 (4.2)	.998	13 (2.2)	1 (0.7)	.255
CAD	629 (8.8)	168 (9.1)	.717	605 (9.2)	163 (9.5)	.719	24 (4.0)	5 (3.6)	.809
AF	287 (4.0)	87 (4.7)	.202	280 (4.3)	85 (5.0)	.229	7 (1.2)	2 (1.4)	.802
Liver disease	226 (3.1)	42 (2.3)	.055	217 (3.3)	40 (2.3)	.049	9 (1.5)	2 (1.4)	.947
Pneumonia	484 (6.7)	159 (8.6)	.007	484 (7.4)	159 (9.3)	.009	0 (0.0)	0 (0.0)	NA
CHF	684 (9.5)	193 (10.4)	.270	661 (10.0)	186 (10.9)	.346	23 (3.9)	7 (5.0)	.533
Endocarditis	11 (0.2)	3 (0.2)	1.000	11 (0.2)	3 (0.2)	1.000	0 (0.0)	0 (0.0)	NA
Personal history of irradiation	1460 (20.3)	313 (16.9)	.001	1407 (21.4)	308 (18.0)	.002	53 (8.9)	5 (3.6)	.036
Discharge quarter									
Jan.–Mar.	1757 (24.5)	426 (23.0)	.546	1611 (24.5)	391 (22.8)	.547	146 (24.6)	35 (25.2)	.846
Apr.–Jun.	1731 (24.1)	462 (24.9)		1599 (24.3)	428 (25.0)		132 (22.2)	34 (24.5)	
Jul.–Sep.	1772 (24.7)	455 (24.6)		1602 (24.3)	421 (24.6)		170 (28.6)	34 (24.5)	
Oct.–Dec.	1917 (26.7)	510 (27.5)		1771 (26.9)	474 (27.7)		146 (24.6)	36 (25.9)	
Hospital bed size‖									
Small	1007 (14.0)	223 (12.0)	.038	936 (14.2)	207 (12.1)	.037	71 (12.0)	16 (11.5)	.655
Medium	1726 (24.0)	431 (23.3)		1579 (24.0)	399 (23.3)		147 (24.7)	32 (23.0)	
Large	4444 (61.9)	1199 (64.7)		4068 (61.8)	1108 (64.6)		376 (63.3)	91 (65.5)	
Hospital location									
Large metropolitan areas	5370 (74.8)	895 (48.3)	<.001	4927 (74.8)	817 (47.7)	<.001	443 (74.6)	78 (56.1)	.031
Small metropolitan areas	1807 (25.2)	656 (35.4)		1656 (25.2)	619 (36.1)		151 (25.4)	37 (26.6)	
Micropolitan areas	0 (0.0)	231 (12.5)		0 (0.0)	209 (12.2)		0 (0.0)	22 (15.8)	
Not metropolitan or micropolitan	0 (0.0)	71 (3.8)		0 (0.0)	69 (4.0)		0 (0.0)	2 (1.4)	
Ownership									
Government, nonfederal	969 (13.5)	163 (8.8)	<.001	889 (13.5)	148 (8.6)	<.001	80 (13.5)	15 (10.8)	.008
Private, nonprofit	5715 (79.6)	1391 (75.1)		5221 (79.3)	1280 (74.7)		494 (83.2)	111 (79.9)	
Private, invest-own	493 (6.9)	299 (16.1)		473 (7.2)	286 (16.7)		20 (3.4)	13 (9.4)	

AF = atrial fibrillation, AKI = acute kidney injury, BC = breast cancer, CAD = coronary artery disease, CCI = Charlson comorbidity index, CHF = congestive heart failure, CKD = chronic kidney disease, COPD = chronic obstructive pulmonary disease, IQR = interquartile range, LOS = length of stay, SD = standard deviation.

* Estimated median household income for the patient’s zip code, stratified into quartiles each year.

† Procedure Codes were defined based on CCS procedure category (breast-conserving surgery [166]) from https://hcup-us.ahrq.gov/toolssoftware/ccsr/prccsr.jsp.

‡ Procedure codes were defined based on CCS procedure category: mastectomy (167).

§ Procedure Codes were defined based on CCS procedure category: cancer chemotherapy (224).

‖ Hospital size is based on the number of hospital beds; cut points were chosen within each region and hospital type strata so that approximately one-third of hospitals would appear in each category.

Patients at teaching hospitals were generally younger for both radiotherapy (median [IQR], 61 [51–70] years vs 64 [54–73] years; *P* < .001) and brachytherapy (median [IQR], 59 [49–67] years vs 62 [51–71] years; *P* = .015). These patients were less likely to be covered by Medicare but had higher rates of medicaid and private insurance. Radiotherapy patients at teaching hospitals had higher rates of metastatic cancer (52.1% vs 46.3%, *P* < .001) and chemotherapy use (2.8% vs 0.6%, *P* < .001) but lower rates of breast-conserving surgery (4.9% vs 7.1%, *P* = .001) and mastectomy (20.0% vs 22.3%, *P* = .036) compared to those at non-teaching hospitals. Additionally, these patients exhibited a greater prevalence of obesity (10.0% vs 7.8%, *P* = .008) and liver disease (3.3% vs 2.3%, *P* = .049), and a higher prevalence of previous irradiation history (21.4% vs 18.0%, *P* = .002). Among patients receiving brachytherapy, no significant differences in comorbidity burden or specific baseline comorbidities were observed. Teaching hospitals typically had fewer beds, were predominantly located in metropolitan areas, and were more likely to be government-controlled. Baseline characteristics of breast cancer patients stratified by hospital teaching status are provided in Table S3, Supplemental Digital Content, https://links.lww.com/MD/Q51.

### 3.3. Clinical outcomes

Table 3 illustrates the clinical outcomes stratified by treatment types and hospital teaching status. Patients receiving radiotherapy exhibited higher rates of adverse outcomes, including in-hospital mortality, complications, and readmissions, compared to those receiving brachytherapy. The in-hospital mortality rate for breast cancer patients was significantly higher in those receiving radiotherapy compared to brachytherapy (4.2% vs 0.3%). However, there were no statistically significant differences between teaching and non-teaching hospitals for either treatment modality (radiotherapy: 4.3% vs 3.7%; *P* = .343; brachytherapy: 0.3% vs 0.0%; *P* = .494). For complications, the prevalence was higher in non-teaching hospitals than in teaching hospitals for both treatment types (radiotherapy: 43.0% vs 45.5%; *P* = .047; brachytherapy: 12.1% vs 18.0%; *P* = .066), although the *P*-value indicates that the difference in brachytherapy patients between hospital teaching status was not statistically significant. Among patients receiving radiotherapy, those in non-teaching hospitals had the highest rate of radiation-related complications, while those receiving brachytherapy in teaching hospitals had the lowest (45.5% vs 12.1%). Regarding readmissions, there were significant differences based on hospital teaching status. Patients receiving radiotherapy alone in teaching hospitals had higher 30- and 90-day readmission rates compared to those in non-teaching hospitals (30-day readmission: 17.1% vs 14.5%, *P* = .009; 90-day readmission: 26.2% vs 22.9%, *P* = .006). For patients receiving brachytherapy, readmission rates were slightly higher at non-teaching hospitals; however, this difference was not statistically significant (*P* > .05).

**Table 3:** Outcomes of in-hospital mortality, readmission and complications for BC patients receiving radiotherapy vs brachytherapy stratified by hospital teaching status, 2016–2021.

	Overall			Radiotherapy-only			Brachytherapy ± radiotherapy		
	Teaching hospital (N = 7177	Non-teaching hospital (N = 1853)	*P*-value	Teaching hospital (N = 6583)	Non-teaching hospital (N = 1714)	*P*-value	Teaching hospital (N = 594)	Non-teaching hospital (N = 139)	*P*-value
Outcomes	n (%)	n (%)		n (%)	n (%)		n (%)	n (%)	
In-hospital mortality	284 (4.0)	64 (3.5)	.349	282 (4.3)	64 (3.7)	.343	2 (0.3)	0 (0.0)	.494
Radiation-related complication	2900 (40.4)	805 (43.4)	.019	2828 (43.0)	780 (45.5)	.047	72 (12.1)	25 (18.0)	.066
Readmission outcomes									
30-d readmission	1159 (16.1)	257 (13.9)	.018	1128 (17.1)	248 (14.5)	.009	31 (5.2)	9 (6.5)	.558
90-d readmission	1779 (24.8)	411 (22.2)	.021	1727 (26.2)	393 (22.9)	.006	52 (8.8)	18 (12.9)	.130

Radiation-related complication, include radiation-induced wounds, infections, strokes, thrombotic events, gastrointestinal issues, cardiotoxicity and respiratory diseases, neurological disorders, skin conditions, pulmonary diseases, lymphedema, shoulder joint disorders, or unspecified conditions, patients with any of these were classified as having a radiation-related complication; Readmission, readmission analyses consider the time between the end of one admission and the start on the next admission.

BC = breast cancer.

### 3.4. Procedure-based outcomes

Table 4 presents unadjusted and adjusted clinical outcomes derived from logistic regression models, including in-hospital mortality, complications, and readmissions.

**Table 4:** Unadjusted and adjusted clinical outcomes of in-hospital mortality, readmission and complications stratified by hospital teaching status, from 2016 to 2021.

	Model 1*	Model 2†	Model 3‡	Model 4§	Model 5‖	Model 6¶
	OR (95% CI)	OR (95% CI)	OR (95% CI)	OR (95% CI)	OR (95% CI)	OR (95% CI)
Overall						
In-hospital mortality	0.87 (0.66, 1.14)	0.86 (0.65, 1.13)	0.95 (0.72, 1.26)	0.85 (0.63, 1.14)	0.88 (0.64, 1.22)	0.91 (0.69, 1.22)
Radiation-related complication	1.13 (1.02, 1.26)	1.12 (1.01, 1.24)	1.20 (1.07, 1.34)	0.92 (0.87, 0.98)	1.07 (0.94, 1.23)	1.13 (0.99, 1.28)
Readmission outcomes						
30-d readmission	0.84 (0.72, 0.97)	0.85 (0.73, 0.99)	0.89 (0.77, 1.03)	0.83 (0.71, 0.97)	0.94 (0.79, 1.10)	0.90 (0.78, 1.05)
90-d readmission	0.86 (0.77, 0.98)	0.87 (0.77, 0.98)	0.92 (0.81, 1.04)	0.86 (0.75, 0.98)	0.96 (0.83, 1.10)	0.93 (0.82, 1.05)
Radiotherapy-only						
In-hospital mortality	0.87 (0.66, 1.14)	0.85 (0.65, 1.13)	0.96 (0.73, 1.27)	0.85 (0.63, 1.15)	0.89 (0.64, 1.23)	0.93 (0.70, 1.23)
Radiation-related complication	1.11 (1.01, 1.21)	1.09 (0.98, 1.22)	1.18 (1.05, 1.32)	0.99 (0.88, 1.11)	1.06 (0.92, 1.22)	1.12 (0.98, 1.26)
Readmission outcomes						
30-d readmission	0.82 (0.70, 0.95)	0.83 (0.72, 0.97)	0.88 (0.75, 1.02)	0.81 (0.69, 0.95)	0.92 (0.78, 1.09)	0.89 (0.77, 1.04)
90-d readmission	0.84 (0.74, 0.95)	0.84 (0.74, 0.95)	0.89 (0.79, 1.02)	0.83 (0.73, 0.95)	0.93 (0.81, 1.07)	0.89 (0.78, 1.02)
Brachytherapy ± radiotherapy						
In-hospital mortality	0.00 (0.00, Inf)	0.00 (0.00, Inf)	0.00 (0.00, Inf)	0.00 (0.00, Inf)	0.77 (0.00, Inf)	0.00 (0.00, Inf)
Radiation-related complication	1.59 (0.97, 2.62)	1.48 (0.89, 2.45)	1.58 (0.95, 2.61)	1.31 (0.77, 2.24)	1.67 (0.87, 3.21)	1.54 (0.87, 2.70)
Readmission outcomes						
30-d readmission	1.26 (0.58, 2.71)	1.25 (0.58, 2.70)	1.22 (0.57, 2.64)	1.37 (0.62, 3.03)	1.55 (0.58, 4.16)	1.21 (0.56, 2.63)
90-d readmission	1.55 (0.88, 2.74)	1.49 (0.84, 2.65)	1.55 (0.88, 2.75)	1.50 (0.84, 2.66)	1.85 (0.94, 3.66)	1.58 (0.89, 2.81)

Radiation-related complication, include radiation-induced wounds, infections, strokes, thrombotic events, gastrointestinal issues, cardiotoxicity and respiratory diseases, neurological disorders, skin conditions, pulmonary diseases, lymphedema, shoulder joint disorders, or unspecified conditions, patients with any of these were classified as having a radiation-related complication; Readmission, readmission analyses consider the time between the end of one admission and the start on the next admission.

AIC = Akaike information criterion, AKI = acute kidney injury, CKD = chronic kidney disease, OR = odds ratio.

* Unadjusted original logistic regression model.

† Adjusted for both age and year.

‡ Adjusted for clinical characteristics (including Metastatic cancer, breast conservatory surgery, mastectomy and chemotherapy).

§ Adjusted for hospital characteristics (including hospital bed size, hospital location and hospital ownership).

‖ Fully adjusted for age, year, baseline comorbidities (including COPD, depression, diabetes, hypertension, hypothyroidism, alcohol abuse, smoker, obesity, anemic-deficiency, coagulopathy, neutropenia, nausea and vomiting, metabolic disorders, AKI, CKD, coronary artery disease, atrial fibrillation, liver disease, pneumonia, congestive, endocarditis), personal history of irradiation, clinical characteristics (including metastatic cancer, breast conservatory surgery, mastectomy and chemotherapy) and hospital characteristics (including hospital bed size, hospital location and hospital ownership).

¶ Model optimized from Model 5 through stepwise regression, with final adjusted variables selected based on the optimal AIC; see Table S13 (Supplemental Digital Content, https://links.lww.com/MD/Q51) for detailed variable selection.

In the unadjusted model, in-hospital mortality appeared lower for patients receiving radiotherapy at non-teaching hospitals compared with teaching hospitals; however, this difference was not statistically significant, as the 95% confidence interval for the odds ratio (0.87 [0.66, 1.14]) included 1. After adjustment for age, year, and clinical and hospital characteristics, the adjusted odds ratios remained stable but were not statistically significant (*P* > .05). Stepwise regression analysis (model 6) yielded consistent results, with no significant association between hospital teaching status and in-hospital mortality (OR [95% CI], 0.93 [0.70, 1.23]). Due to the small number of events, the odds ratio for in-hospital mortality among patients receiving brachytherapy at non-teaching hospitals could not be reliably estimated.

For patients receiving radiotherapy, non-teaching hospitals initially appeared to be positively associated with radiation-related complications. However, after adjustment for hospital characteristics, the likelihood of complications was slightly lower at non-teaching hospitals compared with teaching hospitals, but this difference was not statistically significant (OR [95% CI]: 0.99 [0.88, 1.11]). In stepwise regression, the findings remained consistent (OR [95% CI], 1.12 [0.98, 1.26]), further supporting the lack of association between hospital teaching status and complication risk. Among patients undergoing brachytherapy, treatment at a non-teaching hospital was not significantly associated with an increased risk of radiation-related complications (OR [95% CI], 1.59 [0.97, 2.62]). This result also remained nonsignificant in the stepwise model (OR [95% CI], 1.54 [0.87, 2.70]). In unadjusted models, patients receiving radiotherapy at non-teaching hospitals had lower 30- and 90-day readmission rates compared with those at teaching hospitals (30-day: OR [95% CI], 0.82 [0.70–0.95]; 90-day: OR [95% CI], 0.84 [0.74–0.95]). However, after adjustment for clinical characteristics and in fully adjusted models, hospital teaching status was not significantly associated with readmission rates among radiotherapy patients. Stepwise regression produced similar findings, with no significant association between teaching status and 30-day (OR [95% CI], 0.89 [0.77, 1.04]) or 90-day readmissions (OR [95% CI], 0.89 [0.78, 1.02]). For patients receiving brachytherapy at non-teaching hospitals, both 30- and 90-day readmission rates appeared higher compared to those at teaching hospitals (30-day readmission: OR [95% CI]: 1.26 [0.58, 2.71]; 90-day readmission: OR [95% CI]: 1.55 [0.88, 2.74]). After adjustment for hospital characteristics, the odds of 30- and 90-day readmission were higher for patients at non-teaching hospitals (30-day: OR [95% CI], 1.55 [0.58, 4.16]; 90-day: OR [95% CI], 1.85 [0.94, 3.66]); however, none of these differences reached statistical significance, as the confidence intervals included 1. Results from the stepwise regression model were consistent with the adjusted models, showing no statistically significant differences (30-day: OR [95% CI], 1.21 [0.56, 2.63]; 90-day: OR [95% CI], 1.58 [0.89, 2.81]). Overall, among patients undergoing brachytherapy, readmission rates did not differ significantly by hospital teaching status.

Model comparisons using the AIC demonstrated that the stepwise regression model (model 6) had the lowest AIC value, indicating superior model fit while retaining key covariates. Importantly, across all outcomes, the results derived from the stepwise model were highly consistent with those from the fully adjusted models, reinforcing the robustness and internal validity of the findings.

Detailed multivariable regression estimates, AIC values, and final variable selections from the stepwise regression models are presented in Tables S4–S10, S12, and S13, Supplemental Digital Content, https://links.lww.com/MD/Q51.

### 3.5. Costs and healthcare resource utilization

Table S11, Supplemental Digital Content, https://links.lww.com/MD/Q51 presents LOS and costs for both the radiotherapy and brachytherapy cohorts, stratified by hospital teaching status.

During the first hospitalization, breast cancer patients undergoing radiotherapy had a longer median LOS compared to those receiving brachytherapy (LOS, median [IQR]: 4 days [2–8] vs 2 days [1–3]), and incurred higher average costs (Cost, Mean [SD]: $25,624.45 [$35,472.50] vs $22,622.66 [$18,651.87]; Table S2, Supplemental Digital Content, https://links.lww.com/MD/Q51). In teaching hospitals, the average costs for radiotherapy and brachytherapy patients during their first hospitalization were similar. However, in non-teaching hospitals, the median cost for brachytherapy patients was higher than for radiotherapy patients (cost, in 1000 US dollars, median [IQR]: 17.6 [10.5–29.7] vs 13.9 [7.3–26.8]).

Multivariable logistic regression analysis revealed that among patients receiving radiotherapy, treatment at non-teaching hospitals was associated with lower median costs and shorter LOS. The difference in LOS remained statistically significant after adjustment for potential confounders. In contrast, the difference in average hospitalization costs between teaching and non-teaching hospitals decreased after adjustment and was further attenuated in the fully adjusted model, ultimately becoming statistically non-significant (median net change in LOS [days, 95% CI]: −0.3 [−1.1 to 0.5]). For brachytherapy patients, those in non-teaching hospitals had shorter LOS but higher average costs compared to those in teaching hospitals. After adjustment for potential confounders, these results remained consistent, although the differences did not reach statistical significance.

## 4. Discussion

This retrospective study provides critical insights into how educational factors at medical institutions affect the prognosis and health care resource utilization of breast cancer patients undergoing radiotherapy and brachytherapy. Our analysis revealed that patients treated at teaching hospitals were more likely to receive radiotherapy and brachytherapy, were younger, and had lower Medicare coverage, yet experienced longer hospital stays. These patients also had higher rates of Medicaid and private insurance, higher prevalence of obesity, liver disease, and prior irradiation, and were more likely to have metastatic cancer and receive chemotherapy, but had lower rates of breast-conserving surgery and mastectomy compared with patients at non-teaching hospitals, as shown in the baseline characteristics. Additionally, although patients receiving radiotherapy at non-teaching hospitals initially appeared to be at higher risk for radiation-related complications and less likely to require readmission within 30 to 90 days after discharge, adjustment for confounders rendered these differences not statistically significant. Furthermore, teaching hospital status was not significantly associated with severe adverse events such as in-hospital mortality. Among patients receiving brachytherapy, teaching hospital status was not significantly associated with rates of in-hospital adverse events, complications, or readmissions. No significant differences in comorbidity burden or specific baseline comorbidities were observed among brachytherapy patients between hospital types. This suggests that while the institutional educational environment influences certain aspects of breast cancer care, its impact varies between different treatment modalities.

This study also reveals that, over time, the proportion of patients receiving radiotherapy has increased, while the proportion undergoing brachytherapy has declined. This trend was especially pronounced in teaching hospitals, where radiotherapy rates rose from 63.2% in 2016 to 78.2% in 2021, while both radiotherapy and brachytherapy proportions declined in non-teaching hospitals. In a National Cancer Database (NCDB) analysis, researchers observed a steep decline in brachytherapy for breast cancer APBI since 2008, with a slight increase in intraoperative radiotherapy (IORT) and external beam radiotherapy (EBRT).^[11]^ The decline in brachytherapy is attributed to its higher invasiveness and lower convenience compared with other APBI treatments.^[12]^ Consistent with our findings, the NCDB study noted that brachytherapy patients tend to be younger and healthier, and this subset is often more willing to undergo invasive treatment modalities. Additionally, fewer patients at academic hospitals received brachytherapy, likely due to a preference for newer radiotherapy modalities such as IORT, EBRT, and HF-WBI in these institutions.^[11,13]^ Another study on radiotherapy has highlighted that most patients receive treatment in urban teaching hospitals, likely due to the high costs associated with radiotherapy facilities, which are typically available only in larger hospitals. Consequently, patients often seek treatment in these urban centers.^[14,15]^ In our study, approximately 80% of breast cancer patients were treated in teaching hospitals, explaining the higher overall rates of radiotherapy and the decreased proportion in non-teaching settings. The significant patient concentration in teaching hospitals can be attributed to inter-hospital referral mechanisms and the specialized care they offer. When patients present with complex or severe conditions that cannot be adequately managed at their initial hospitals, teaching hospitals serve as referral centers.^[16–18]^

Previous studies have noted that the prevalence of postoperative complications in teaching hospitals is similar to, or even higher than, that in non-teaching hospitals.^[19–22]^ However, our study revealed that the prevalence of radiation-related complications in patients receiving radiotherapy was higher in non-teaching hospitals than in teaching hospitals (45.5% vs 43.0%, *P* = .047), although this difference was not statistically significant after adjustment (OR, 0.99; 95% CI, 0.88, 1.11), and unadjusted models indicated that patients in non-teaching hospitals had a 1.11 times higher risk of complications. For brachytherapy, complication rates were not significantly different between hospital types. Our findings suggest that, despite earlier research showing no advantage for teaching hospitals in surgical complication rates, teaching hospitals demonstrate superior outcomes in radiotherapy for breast cancer patients. This may be attributed to a combination of academic and clinical factors. Teaching hospitals typically possess more advanced radiotherapy equipment and technology. By integrating medical imaging with radiation delivery systems, they enhance treatment precision, reducing radiation exposure to healthy tissue and lowering complication risks.^[23]^ Recent studies on radiotherapy training emphasize the shift from traditional apprenticeship models to more specialized training and highlight the importance of specialized education in oncologic radiotherapy.^[24]^ Teaching hospitals have a distinct advantage in providing such training.^[25]^ They offer comprehensive training programs where medical staff undergo rigorous and continuous professional development, with opportunities to engage in research and educational activities. Evidence suggests that staff at major teaching hospitals adhere more closely to guidelines, particularly in low-tech care settings.^[26]^ Additionally, patient management and care in teaching hospitals are often more systematic and refined, with higher overall care quality, especially in cognitive skills and diagnostics.^[27]^ This enables health care professionals to promptly identify and address adverse reactions during treatment. The resource allocation advantages in teaching hospitals, along with their leadership in adopting new technologies and treatment modalities, may further reduce the prevalence of radiotherapy-related complications. These findings highlight the potential advantages of teaching hospitals in radiotherapy and suggest that the characteristics and resources of teaching hospitals may differently impact patient outcomes across various treatment modalities. This insight provides a perspective for future exploration of the role and effectiveness of teaching hospitals in diverse therapeutic contexts.

In breast cancer patients undergoing radiotherapy, hospitalization costs and LOS are significantly higher for patients treated at teaching hospitals compared with non-teaching hospitals. Our study showed that the average expenditure for patients treated at teaching hospitals is 1.27 times that of those at non-teaching hospitals, and the average LOS is 1.20 times longer. Increased hospitalization costs in teaching hospitals may be associated with higher operating costs. One study indicates that major teaching hospitals incur costs approximately 10 to 20% higher than non-teaching hospitals.^[26]^ Baseline characteristics of hospitals treating radiotherapy patients revealed that 74.8% of teaching hospitals were located in metropolitan areas, compared with only 47.7% of non-teaching hospitals. Higher operating costs and the expense of resource-intensive services in large cities could be contributing factors to increased hospitalization costs in teaching hospitals.

Moreover, the increased expenditure may also be due to the more complex and severe conditions of patients admitted to teaching hospitals, necessitating higher levels of care and more complex treatment plans.^[28]^ According to the risk coordination effect theory, patients in teaching hospitals face higher risks compared with those in non-teaching hospitals, leading to increased resource utilization and costs.^[29]^ However, after adjustment for hospital characteristics, clinical features, and complications, no significant differences in median hospitalization costs or LOS were observed between patients receiving radiotherapy at teaching and non-teaching hospitals (median net change in LOS [days, 95% CI]: −0.3 [−1.1–0.5]). These findings suggest that, after controlling for key confounding factors, teaching hospital status does not significantly influence hospitalization costs or LOS for radiotherapy patients.

For breast cancer patients undergoing brachytherapy, there are no significant differences in hospitalization lengths and costs between teaching and non-teaching hospitals, either before or after adjustment. Our results indicate that patients receiving brachytherapy at different teaching hospitals had balanced baseline economic conditions, insurance types, clinical characteristics, and comorbidities. The similarity in clinical features among patients results in consistent treatment needs and resource utilization, reducing disparities between hospital types. We also found that non-teaching hospitals had slightly shorter LOS but higher average costs for brachytherapy, yet these differences were not statistically significant after adjustment.

Although teaching hospitals have higher operating costs,^[26]^ in the context of brachytherapy, a specific, short-term treatment,^[30]^ cost-control measures can minimize differences in expenses. Furthermore, the relatively simple condition of patients, lower treatment complexity, and high standardization of procedures are likely reasons for the lack of significant differences in hospitalization costs and LOS for breast cancer patients undergoing brachytherapy at different teaching hospitals. Brachytherapy is primarily indicated for early-stage breast cancer patients. Accelerated partial breast irradiation (APBI) based on brachytherapy is the only treatment with Level 1 evidence as an effective alternative to whole breast irradiation following breast-conserving surgery for low-risk early-stage breast cancer.^[31–33]^ Consequently, patients receiving brachytherapy are mostly in early stages, with smaller tumor sizes and fewer complex metastases, making treatment relatively straightforward. Brachytherapy itself is a highly standardized technique, requiring adherence to strict clinical guidelines and protocols.^[34]^ Thus, regardless of whether the treatment is administered in teaching or non-teaching hospitals, the equipment, techniques, and treatment plans are typically consistent. Overall, our findings indicate that outcomes and resource utilization for brachytherapy are similar across hospital types.

This study found that among breast cancer patients receiving radiotherapy, both clinical outcomes and treatment costs differed significantly between teaching and non-teaching hospitals. In contrast, no significant differences in outcomes were observed among patients receiving brachytherapy across hospital types. These findings suggest that the impact of hospital teaching status on patient outcomes and healthcare resource utilization may vary depending on the treatment modality. However, further investigation is needed to better understand the specific impact of educational factors on patient outcomes. One key area for exploration involves the educational interventions and training programs within teaching hospitals that may enhance the effectiveness of radiotherapy. These could include advancements in radiotherapy techniques, the development of personalized treatment plans, and optimal practices for multidisciplinary collaboration. Identifying such strategies could provide valuable insights for broader application across health care systems, promoting practices that improve patient outcomes. Another important consideration is the impact of teaching hospital status on the long-term quality of life and mental health of breast cancer patients after treatment. Investigating these aspects could reveal crucial care dimensions beyond standard clinical outcomes.^[35,36]^ Moreover, while much of the existing research has concentrated on the influence of teaching hospital status on surgical outcomes,^[21,37]^ our findings indicate that different educational environments may affect treatment modalities differently. Given the multifaceted nature of breast cancer therapy, future research should focus on how educational resources within teaching hospitals shape the effectiveness of various treatment approaches. Such insights could guide the development of more effective, integrated treatment strategies tailored to individual patient needs.

## 5. Limitation

A major strength of this study is the use of a study population drawn from the NRD, one of the largest and most ethnically diverse inpatient datasets in the United States. This robust dataset enhances the external validity of our findings by capturing both initial and recurrent hospitalizations across diverse demographic groups nationwide. However, several limitations of this study must be acknowledged. First, the study is based on administrative data from the NRD and employs a retrospective design, which inherently carries the risk of unmeasured confounding. This limitation restricts the ability to draw causal inferences. Second, detailed clinical characteristics of breast cancer patients were difficult to capture using the available data, limiting our ability to comprehensively adjust for disease severity, possibly influencing the precision and interpretability of our findings. In addition, reliance on ICD coding introduces the potential for misclassification or variability in coding practices across institutions. The accuracy of the dataset depends on the consistency and reliability of coding at each participating hospital, which may lead to discrepancies. Moreover, the limited granularity of ICD-10-CM diagnostic codes makes it challenging to ascertain the exact clinical indications for radiotherapy or brachytherapy in individual patients. For instance, although we included patients hospitalized after a breast cancer diagnosis who received radiotherapy or brachytherapy, we were unable to determine the specific indications for these treatments due to the limitations of ICD coding. Furthermore, due to the limitations of diagnostic coding, we were unable to reliably distinguish patients who received brachytherapy alone from those who received both brachytherapy and external beam radiotherapy. As a result, the “brachytherapy ± radiotherapy” cohort may include patients who underwent both treatments, which introduces potential heterogeneity within this group. This coding-related constraint limited our ability to perform a separate analysis on brachytherapy-only patients and may have influenced the observed outcomes in that cohort. This limitation should be considered when interpreting differences in outcomes between the 2 treatment groups. Furthermore, this study did not account for several potential mediating factors that could influence the relationship between hospital teaching status and patient outcomes, including institutional financial capacity, facility availability, and access to specialized personnel. These unmeasured variables may partially explain differences in care quality and should be explored in future research. Another important limitation is that the NRD captures only inpatient data and does not include breast cancer patients treated with radiotherapy or brachytherapy in the outpatient setting. While these therapies are typically administered in inpatient environments due to equipment and monitoring requirements, outpatient treatments – especially for patients with early-stage disease and simpler regimens – do occur. These patients may differ from inpatients in terms of baseline characteristics and treatment contexts. As such, excluding outpatient data may introduce selection bias and limit the applicability of our findings to the broader breast cancer population, particularly those receiving outpatient care. Because of this limitation, we were also unable to evaluate the impact of hospital teaching status on treatment outcomes among outpatient populations, which may affect the overall comprehensiveness of our conclusions. Future studies should integrate both inpatient and outpatient data to provide a more complete understanding of how hospital teaching status influences breast cancer treatment outcomes. Nonetheless, given that this study focuses on hospitalized patients and that breast cancer radiotherapy and brachytherapy are most commonly administered in inpatient settings, the exclusion of outpatient cases is unlikely to substantially compromise the robustness of our primary conclusions. However, this limitation should be taken into account when interpreting the results.

## 6. Conclusions

Hospital teaching status significantly affects prognosis and health care resource utilization in breast cancer patients undergoing radiotherapy, but is not significantly associated with outcomes or resource use in those receiving brachytherapy. These findings suggest that variations in teaching environments may differentially impact treatment modalities. Given the complexity of breast cancer management, future research should examine how educational resources in teaching hospitals influence the efficacy of different treatment approaches to inform the development of more effective, individualized strategies.Supplemental digital contents “Tables S14 and S15” are available for this article (https://links.lww.com/MD/Q51).

## Author contributions

**Conceptualization:** Jifang Zhou, Fan Yang.

**Data curation:** Yimei Zhong.

**Formal analysis:** Yimei Zhong, Bingxin Liu.

**Methodology:** Yimei Zhong, Jifang Zhou, Fan Yang.

**Supervision:** Bingxin Liu.

**Validation:** Yimei Zhong, Bingxin Liu.

**Visualization:** Yimei Zhong.

**Writing – original draft:** Yimei Zhong.

**Writing – review & editing:** Jifang Zhou, Yali Ge, Na Rui, Liliang Shen, Fan Yang.

## Supplementary Material
