# Supplementary material for: The effect of hospital teaching status on adverse outcomes among breast cancer patients receiving radiotherapy and brachytherapy
Source: Medicine (Baltimore). 2025 Oct 3;104(40):e44648. doi: 10.1097/MD.0000000000044648 (PMC12499757; doi:10.1097/MD.0000000000044648)
Supplement: Supplementary file 1 [file medi-104-e44648-s001.docx]

**Table S1.** Diagnosis and procedure codes used in the present study in condition ascertainment.

|  | **ICD-10 codes** |
| --- | --- |
| Breast cancer | C50.01X, C50.11X, C50.21ZX, C50.31X, C50.41X, C50.51X, C50.61X, C50.81X, C50.91X, D05.0X, D05.1X |
| **Comorbidities** | |
| Chronic Obstructive Pulmonary Disease | I278, I279, J40, J41, J42, J43, J44, J45, J46, J47, J60, J61, J62', J63, J64, J65, J66, J67, J684, J701, J703 |
| Diabetes | E100, E101, E106, E108, E109, E110, E111, E116, E118, E119, E120, E121, E126, E128, E129, E130, E131, E136, E138, E139, E140, E141, E146, E148, E149 |
| Hypertension | I10.x, I11.0, I11.9 |
| Obesity | E66.0X, E66.1, E66.2, E66.3, E66.8, E66.9 |
| Alcohol Use | [F101](https://www.icd10data.com/ICD10CM/Codes/F01-F99/F10-F19/F10-/F10.1), [F1010](https://www.icd10data.com/ICD10CM/Codes/F01-F99/F10-F19/F10-/F10.10), [F1011](https://www.icd10data.com/ICD10CM/Codes/F01-F99/F10-F19/F10-/F10.11), [F1012](https://www.icd10data.com/ICD10CM/Codes/F01-F99/F10-F19/F10-/F10.12), [F10120](https://www.icd10data.com/ICD10CM/Codes/F01-F99/F10-F19/F10-/F10.120), [F10121](https://www.icd10data.com/ICD10CM/Codes/F01-F99/F10-F19/F10-/F10.121), [F10129](https://www.icd10data.com/ICD10CM/Codes/F01-F99/F10-F19/F10-/F10.129), [F1014](https://www.icd10data.com/ICD10CM/Codes/F01-F99/F10-F19/F10-/F10.14), [F1015](https://www.icd10data.com/ICD10CM/Codes/F01-F99/F10-F19/F10-/F10.15), [F10150](https://www.icd10data.com/ICD10CM/Codes/F01-F99/F10-F19/F10-/F10.150), [F10151](https://www.icd10data.com/ICD10CM/Codes/F01-F99/F10-F19/F10-/F10.151), [F10159](https://www.icd10data.com/ICD10CM/Codes/F01-F99/F10-F19/F10-/F10.159), [F1018](https://www.icd10data.com/ICD10CM/Codes/F01-F99/F10-F19/F10-/F10.18), [F10180](https://www.icd10data.com/ICD10CM/Codes/F01-F99/F10-F19/F10-/F10.180), [F10181](https://www.icd10data.com/ICD10CM/Codes/F01-F99/F10-F19/F10-/F10.181), [F10182](https://www.icd10data.com/ICD10CM/Codes/F01-F99/F10-F19/F10-/F10.182), [F10188](https://www.icd10data.com/ICD10CM/Codes/F01-F99/F10-F19/F10-/F10.188), [F1019](https://www.icd10data.com/ICD10CM/Codes/F01-F99/F10-F19/F10-/F10.19), [F102](https://www.icd10data.com/ICD10CM/Codes/F01-F99/F10-F19/F10-/F10.2), [F1020](https://www.icd10data.com/ICD10CM/Codes/F01-F99/F10-F19/F10-/F10.20), [F1021](https://www.icd10data.com/ICD10CM/Codes/F01-F99/F10-F19/F10-/F10.21), [F1022](https://www.icd10data.com/ICD10CM/Codes/F01-F99/F10-F19/F10-/F10.22), [F10220](https://www.icd10data.com/ICD10CM/Codes/F01-F99/F10-F19/F10-/F10.220), [F10221](https://www.icd10data.com/ICD10CM/Codes/F01-F99/F10-F19/F10-/F10.221), [F10229](https://www.icd10data.com/ICD10CM/Codes/F01-F99/F10-F19/F10-/F10.229), [F1023](https://www.icd10data.com/ICD10CM/Codes/F01-F99/F10-F19/F10-/F10.23), [F10230](https://www.icd10data.com/ICD10CM/Codes/F01-F99/F10-F19/F10-/F10.230), [F10231](https://www.icd10data.com/ICD10CM/Codes/F01-F99/F10-F19/F10-/F10.231), [F10232](https://www.icd10data.com/ICD10CM/Codes/F01-F99/F10-F19/F10-/F10.232), [F10239](https://www.icd10data.com/ICD10CM/Codes/F01-F99/F10-F19/F10-/F10.239), [F1024](https://www.icd10data.com/ICD10CM/Codes/F01-F99/F10-F19/F10-/F10.24), [F1025](https://www.icd10data.com/ICD10CM/Codes/F01-F99/F10-F19/F10-/F10.25), [F10250](https://www.icd10data.com/ICD10CM/Codes/F01-F99/F10-F19/F10-/F10.250), [F10251](https://www.icd10data.com/ICD10CM/Codes/F01-F99/F10-F19/F10-/F10.251), [F10259](https://www.icd10data.com/ICD10CM/Codes/F01-F99/F10-F19/F10-/F10.259), [F1026](https://www.icd10data.com/ICD10CM/Codes/F01-F99/F10-F19/F10-/F10.26), [F1027](https://www.icd10data.com/ICD10CM/Codes/F01-F99/F10-F19/F10-/F10.27), [F1028](https://www.icd10data.com/ICD10CM/Codes/F01-F99/F10-F19/F10-/F10.28), [F10280](https://www.icd10data.com/ICD10CM/Codes/F01-F99/F10-F19/F10-/F10.280), [F10281](https://www.icd10data.com/ICD10CM/Codes/F01-F99/F10-F19/F10-/F10.281), [F10282](https://www.icd10data.com/ICD10CM/Codes/F01-F99/F10-F19/F10-/F10.282), [F10288](https://www.icd10data.com/ICD10CM/Codes/F01-F99/F10-F19/F10-/F10.288), [F1029](https://www.icd10data.com/ICD10CM/Codes/F01-F99/F10-F19/F10-/F10.29), [F10920](https://www.icd10data.com/ICD10CM/Codes/F01-F99/F10-F19/F10-/F10.920), [F10921](https://www.icd10data.com/ICD10CM/Codes/F01-F99/F10-F19/F10-/F10.921), [F10929](https://www.icd10data.com/ICD10CM/Codes/F01-F99/F10-F19/F10-/F10.929), [F1094](https://www.icd10data.com/ICD10CM/Codes/F01-F99/F10-F19/F10-/F10.94), [F1095](https://www.icd10data.com/ICD10CM/Codes/F01-F99/F10-F19/F10-/F10.95), [F10950](https://www.icd10data.com/ICD10CM/Codes/F01-F99/F10-F19/F10-/F10.950), [F10951](https://www.icd10data.com/ICD10CM/Codes/F01-F99/F10-F19/F10-/F10.951), [F10959](https://www.icd10data.com/ICD10CM/Codes/F01-F99/F10-F19/F10-/F10.959), [F1096](https://www.icd10data.com/ICD10CM/Codes/F01-F99/F10-F19/F10-/F10.96), [F1097](https://www.icd10data.com/ICD10CM/Codes/F01-F99/F10-F19/F10-/F10.97), [F1098](https://www.icd10data.com/ICD10CM/Codes/F01-F99/F10-F19/F10-/F10.98), [F10980](https://www.icd10data.com/ICD10CM/Codes/F01-F99/F10-F19/F10-/F10.980), [F10981](https://www.icd10data.com/ICD10CM/Codes/F01-F99/F10-F19/F10-/F10.981), [F10982](https://www.icd10data.com/ICD10CM/Codes/F01-F99/F10-F19/F10-/F10.982), [F10988](https://www.icd10data.com/ICD10CM/Codes/F01-F99/F10-F19/F10-/F10.988), [F1099](https://www.icd10data.com/ICD10CM/Codes/F01-F99/F10-F19/F10-/F10.99) |
| Smoking | [F17](https://www.icd10data.com/ICD10CM/Codes/F01-F99/F10-F19/F17-/F17), [F172](https://www.icd10data.com/ICD10CM/Codes/F01-F99/F10-F19/F17-/F17.2), [F1720](https://www.icd10data.com/ICD10CM/Codes/F01-F99/F10-F19/F17-/F17.20), [F17200](https://www.icd10data.com/ICD10CM/Codes/F01-F99/F10-F19/F17-/F17.200), [F17201](https://www.icd10data.com/ICD10CM/Codes/F01-F99/F10-F19/F17-/F17.201), [F17203](https://www.icd10data.com/ICD10CM/Codes/F01-F99/F10-F19/F17-/F17.203), [F17208](https://www.icd10data.com/ICD10CM/Codes/F01-F99/F10-F19/F17-/F17.208), [F17209](https://www.icd10data.com/ICD10CM/Codes/F01-F99/F10-F19/F17-/F17.209), [F1721](https://www.icd10data.com/ICD10CM/Codes/F01-F99/F10-F19/F17-/F17.21), [F17210](https://www.icd10data.com/ICD10CM/Codes/F01-F99/F10-F19/F17-/F17.210), [F17211](https://www.icd10data.com/ICD10CM/Codes/F01-F99/F10-F19/F17-/F17.211), [F17213](https://www.icd10data.com/ICD10CM/Codes/F01-F99/F10-F19/F17-/F17.213), [F17218](https://www.icd10data.com/ICD10CM/Codes/F01-F99/F10-F19/F17-/F17.218), [F17219](https://www.icd10data.com/ICD10CM/Codes/F01-F99/F10-F19/F17-/F17.219), [F1722](https://www.icd10data.com/ICD10CM/Codes/F01-F99/F10-F19/F17-/F17.22), [F17220](https://www.icd10data.com/ICD10CM/Codes/F01-F99/F10-F19/F17-/F17.220), [F17221](https://www.icd10data.com/ICD10CM/Codes/F01-F99/F10-F19/F17-/F17.221), [F17223](https://www.icd10data.com/ICD10CM/Codes/F01-F99/F10-F19/F17-/F17.223), [F17228](https://www.icd10data.com/ICD10CM/Codes/F01-F99/F10-F19/F17-/F17.228), [F17229](https://www.icd10data.com/ICD10CM/Codes/F01-F99/F10-F19/F17-/F17.229), [F1729](https://www.icd10data.com/ICD10CM/Codes/F01-F99/F10-F19/F17-/F17.29), [F17290](https://www.icd10data.com/ICD10CM/Codes/F01-F99/F10-F19/F17-/F17.290), [F17291](https://www.icd10data.com/ICD10CM/Codes/F01-F99/F10-F19/F17-/F17.291), [F17293](https://www.icd10data.com/ICD10CM/Codes/F01-F99/F10-F19/F17-/F17.293), [F17298](https://www.icd10data.com/ICD10CM/Codes/F01-F99/F10-F19/F17-/F17.298), [F17299](https://www.icd10data.com/ICD10CM/Codes/F01-F99/F10-F19/F17-/F17.299), Z87.891 |
| Depression | F31.X, F32.X, F33.X, F34.X, F43.21, F43.23 |
| Hypothyroidism | E02, E03.X, E89.0, E89.2 |
| Anemic-deficiency | D50.0, D50.8, D50.9, D51.X, D52.X, D53.X, D61.X, D64.0, D64.1, D64.2, D64.3, D64.4, D64.9, D64.81, D64.89 |
| Coagulopathy | D65-D68.x, D69.1, D69.3-D69.6 |
| Neutropenia | D70.X |
| Nausea and Vomiting | R11.X |
| Metabolic disorders | E70.9, E71.548, E72.9, E73.9, E74.9, E75.6, E76.9, E77.9, E78.9, E80.7, E83.9, E84.9, E85.9, E86.9, E87.8, E88 |
| AKI | N17.9, N19 |
| CKD | N18.32, N18.4, N18.5, N18.6, N18.9, I12.0, Z49.0, Z99.2 |
| Coronary Artery Disease | I09.9, I11.0, I13.0, I13.2, I25.5, I42.0, I42.5-I42.9, I43.x, I50.x, P29.0 |
| Atrial Fibrillation | I48.91, I48.92 |
| Liver Disease | K70.0, K70.1, K70.2, K70.3, K70.4, K70.9, K71.X, K72.X, K73.X, K74.0-K74.69, K75.X, K76.X, K77.X |
| Pneumonia | J18.0, J18.1, J18.2, J18.8, J18.9, J12.X, J13, J14, J15.X, J16.X, J17, J18.X |
| Congestive, CHF | I50, I50.1, I50.20, I50.21, I50.22, I50.23, I50.3, I50.30, I50.31, I50.32, I50.33, I50.4, I50.40, I50.41, I50.42, I50.43, I50.8, I50.81, I50.810, I50.811, I50.812, I50.813, I50.814, I50.82, I50.83, I50.84, I50.89, I50.9 |
| Endocarditis | A39.51, A54.83, B33.21, B37.6, I01.1, I33.0, I33.9, I38, I39 |
| Personal history of irradiation | Z92.3 |
| **Outcome** | |
| Brachytherapy |  |
| Diagnosis Code | Y84.2, Z51.0, W88.1XXA |
| Surgical Procedure Codes | DM000ZZ, DM001ZZ, DM002ZZ, DM003Z0, DM003ZZ, DM004ZZ, DM005ZZ, DM006ZZ, DM010ZZ, DM011ZZ, DM012ZZ, DM013Z0, DM013ZZ, DM014ZZ, DM015ZZ, DM016ZZ, DM20DZZ, DM20HZZ, DM20JZZ, DM21DZZ, DM21HZZ, DM21JZZ, DMY07ZZ, DMY0FZZ, DMY17ZZ, DMY1FZZ, C75LYZZ, CW53YZZ, CW54YZZ, CW56YZZ, CW73YZZ, DB070ZZ, DB071ZZ, DB072ZZ, DB073Z0, DB073ZZ, DB074ZZ, DB075ZZ, DB076ZZ, DB27DZZ, DB27HZZ, DB27JZZ, DBY77ZZ, DBY7FZZ, DH060ZZ, DH061ZZ, DH062ZZ, DH063Z0, DH063ZZ, DH064ZZ, DH065ZZ, DH066ZZ, DHY67ZZ, DHY6FZZ, DW020ZZ, DW021ZZ, DW022ZZ, DW023Z0, DW023ZZ, DW024ZZ, DW025ZZ, DW026ZZ, DW22DZZ, DW22HZZ, DW22JZZ, DWY27ZZ, DWY2FZZ, 0WPB41Z, 0WPBX1Z, 0WW901Z, 0WW931Z, 0WW941Z, 0WW9X1Z, 0WWB01Z, 0WWB31Z, 0WWB41Z, 0WWBX1Z, DB050ZZ, DB051ZZ, DB052ZZ, DB053Z0, DB053ZZ, DB054ZZ, DB055ZZ, DB056ZZ, DB25DZZ, DB25HZZ, DB25JZZ, DBY57ZZ, DBY5FZZ, 0WWC01Z, 0WWC31Z, 0WWC41Z, 0WWCX1Z, DB060ZZ, DB061ZZ, DB062ZZ, DB063Z0, DB063ZZ, DB064ZZ, DB065ZZ, DB066ZZ, DB26DZZ, DB26HZZ, DB26JZZ, DBY67ZZ, DBY6FZZ, DB080ZZ, DB081ZZ, DB082ZZ, DB083Z0, DB083ZZ, DB084ZZ, DB085ZZ, DB086ZZ, DB28DZZ, DB28HZZ, DB28JZZ, DBY87ZZ, DBY8FZZ, DW040ZZ, DW041ZZ, DW042ZZ, DW043Z0, DW043ZZ, DW044ZZ, DW045ZZ, DW046ZZ, DWY47ZZ, DWY4FZZ, CW7NYZZ, DW050ZZ, DW051ZZ, DW052ZZ, DW053Z0, DW053ZZ, DW054ZZ, DW055ZZ, DW056ZZ, DWY57ZZ, DWY5FZZ, DP040ZZ, DP041ZZ, DP042ZZ, DP043Z0, DP043ZZ, DP044ZZ, DP045ZZ, DP046ZZ, DPY47ZZ, DPY4FZZ, D7010ZZ, D7011ZZ, D7012ZZ, D7013Z0, D7013ZZ, D7014ZZ, D7015ZZ, D7016ZZ, D721DZZ, D721HZZ, D721JZZ, D7Y1FZZ, D7050ZZ, D7051ZZ, D7052ZZ, D7053Z0, D7053ZZ, D7054ZZ, D7055ZZ, D7056ZZ, D725DZZ, D725HZZ, D725JZZ, D7Y5FZZ, C755YZZ, C75DYZZ, C75JYZZ, C75KYZZ, C75LYZZ, C75MYZZ, C75NYZZ, C75PYZZ, C75YYZZ, C76YYZZ, D7030ZZ, D7031ZZ, D7032ZZ, D7033Z0, D7033ZZ, D7034ZZ, D7035ZZ, D7036ZZ, D7040ZZ, D7041ZZ, D7042ZZ, D7043Z0, D7043ZZ, D7044ZZ, D7045ZZ, D7046ZZ, D7050ZZ, D7051ZZ, D7052ZZ, D7053Z0, D7053ZZ, D7054ZZ, D7055ZZ, D7056ZZ, D7060ZZ, D7061ZZ, D7062ZZ, D7063Z0, D7063ZZ, D7064ZZ, D7065ZZ, D7066ZZ, D7070ZZ, D7071ZZ, D7072ZZ, D7073Z0, D7073ZZ, D7074ZZ, D7075ZZ, D7076ZZ, D7080ZZ, D7081ZZ, D7082ZZ, D7083Z0, D7083ZZ, D7084ZZ, D7085ZZ, D7086ZZ, D723DZZ, D723HZZ, D723JZZ, D724DZZ, D724HZZ, D724JZZ, D725DZZ, D725HZZ, D725JZZ, D726DZZ, D726HZZ, D726JZZ, D727DZZ, D727HZZ, D727JZZ, D728DZZ, D728HZZ, D728JZZ, D7Y3FZZ, D7Y4FZZ, D7Y5FZZ, D7Y6FZZ, D7Y7FZZ, D7Y8FZZ |
| Radiotherapy |  |
| Surgical Procedure Codes | DM1097Z, DM1098Z, DM1099Z, DM109BZ, DM109CZ, DM109YZ, DM10B6Z, DM10B7Z, DM10B8Z, DM10B9Z, DM10BB1, DM10BBZ, DM10BCZ, DM10BYZ, DM1197Z, DM1198Z, DM1199Z, DM119BZ, DM119CZ, DM119YZ, DM11B6Z, DM11B7Z, DM11B8Z, DM11B9Z, DM11BB1, DM11BBZ, DM11BCZ, DM11BYZ, 0HHT01Z, 0HHT31Z, 0HHT71Z, 0HHT81Z, 0HHTX1Z, 0HHU01Z, 0HHU31Z, 0HHU71Z, 0HHU81Z, 0HHUX1Z, 0HHV01Z, 0HHV31Z, 0HHV71Z, 0HHV81Z, 0HHVX1Z, 0HPT01Z, 0HPT31Z, 0HPT71Z, 0HPT81Z, 0HPTX1Z, 0HPU01Z, 0HPU31Z, 0HPU71Z, 0HPU81Z, 0HPUX1Z, DB1797Z, DB1798Z, DB1799Z, DB179BZ, DB179CZ, DB179YZ, DB17B6Z, DB17B7Z, DB17B8Z, DB17B9Z, DB17BB1, DB17BBZ, DB17BCZ, DB17BYZ, DW1297Z, DW1298Z, DW1299Z, DW129BZ, DW129CZ, DW129YZ, DW12B6Z, DW12B7Z, DW12B8Z, DW12B9Z, DW12BB1, DW12BBZ, DW12BCZ, DW12BYZ, 0WH801Z, 0WH831Z, 0WH841Z, CW73NZZ, CW73YZZ, DB1597Z, DB1598Z, DB1599Z, DB159BZ, DB159CZ, DB159YZ, DB15B6Z, DB15B7Z, DB15B8Z, DB15B9Z, DB15BB1, DB15BBZ, DB15BCZ, DB15BYZ, 0WH901Z, 0WH931Z, 0WHB01Z, 0WHB31Z, 0WH941Z, 0WHB41Z, 3E0L3HZ, DB1697Z, DB1698Z, DB1699Z, DB169BZ, DB169CZ, DB169YZ, DB16B6Z, DB16B7Z, DB16B8Z, DB16B9Z, DB16BB1, DB16BBZ, DB16BCZ, DB16BYZ, 0WHC01Z, 0WHC31Z, 0WHC41Z, DB1897Z, DB1898Z, DB1899Z, DB189BZ, DB189CZ, DB189YZ, DB18B6Z, DB18B7Z, DB18B8Z, DB18B9Z, DB18BB1, DB18BBZ, DB18BCZ, DB18BYZ, CW7N8ZZ, CW7NGZZ, CW7NNZZ, CW7NPZZ, CW7NYZZ, DWY5GDZ, DWY5GFZ, DWY5GGZ, DWY5GHZ, DWY5GYZ, D71197Z, D71198Z, D71199Z, D7119BZ, D7119CZ, D7119YZ, D711B6Z, D711B7Z, D711B8Z, D711B9Z, D711BB1, D711BBZ, D711BCZ, D711BYZ, 07HM01Z, 07HM31Z, 07HM41Z, D71597Z, D71598Z, D71599Z, D7159BZ, D7159CZ, D7159YZ, D715B6Z, D715B7Z, D715B8Z, D715B9Z, D715BB1, D715BBZ, D715BCZ, D715BYZ, D71397Z, D71398Z, D71399Z, D7139BZ, D7139CZ, D7139YZ, D713B6Z, D713B7Z, D713B8Z, D713B9Z, D713BB1, D713BBZ, D713BCZ, D713BYZ, D71497Z, D71498Z, D71499Z, D7149BZ, D7149CZ, D7149YZ, D714B6Z, D714B7Z, D714B8Z, D714B9Z, D714BB1, D714BBZ, D714BCZ, D714BYZ, D71597Z, D71598Z, D71599Z, D7159BZ, D7159CZ, D7159YZ, D715B6Z, D715B7Z, D715B8Z, D715B9Z, D715BB1, D715BBZ, D715BCZ, D715BYZ, D71697Z, D71698Z, D71699Z, D7169BZ, D7169CZ, D7169YZ, D716B6Z, D716B7Z, D716B8Z, D716B9Z, D716BB1, D716BBZ, D716BCZ, D716BYZ, D71797Z, D71798Z, D71799Z, D7179BZ, D7179CZ, D7179YZ, D717B6Z, D717B7Z, D717B8Z, D717B9Z, D717BB1, D717BBZ, D717BCZ, D717BYZ, D71897Z, D71898Z, D71899Z, D7189BZ, D7189CZ, D7189YZ, D718B6Z, D718B7Z, D718B8Z, D718B9Z, D718BB1, D718BBZ, D718BCZ, D718BYZ, 07HN01Z, 07HN31Z, 07HN41Z, 3E0W3HZ |
| Wound (hemorrhage, hematoma, seroma, fat necrosis, wound dehiscence, postoperative fistula) | T81.30XA, T81.32XA, T81.31XA, T81.89XA, T81.83XA, S21.129A, S21.029A, I97.42, I97.620, J95.62, J95.831, L76.01, L76.02, L76.21, L76.22, M96.810, M96.811, M96.830,  M96.831, G97.32, G97.62, I97.42, I97.621, J95.62, J95.861, L76.01, L76.02, L76.31, L76.32, M96.810, M96.811, M96.840, M96.841, G97.64, I97.622, J95.863, L76.33, L76.34,  M96.842, M96.843, N64.1, G97.32, G97.62, I97.418, I97.621, J95.62, J95.861, L76.02, L76.02, G97.64, I97.622, J95.863, L76.34, M96.842, M96.843 |
| Infection (mastitis, abscess, cellulitis, lymphangitis, postoperative infection) | K68.11, T81.83XA, N61.0, N61.1, L03.319, L03.329, L03.119, L03.129, L03.90, L03.91, L08.0, T85.79XA, T81.4XXA |
| Stroke | I97.811, I97.821 |
| Deep venous thrombosis/pulmonary embolism | I26.90, I26.99, T80.0XXA, T81.718A, T81.72XA, T82.817A, T82.818A, I26.99, I80.209, I80.10, I80.3, I80.219, I82.40, I82.419, I82.429, I82.439, I82.4Y9, I82.449, I82.499, I82.4Z9, I82.890, I82.91, Z86.718, T81.719A, T81.72XA, T80.1XXA, T81.718A, T81.72XA, K91.XXX |
| Digestive | K91.XXX, K31.0, R11.10, K52.3, K52.89, K52.9, K56, K56.0, K56.7, K56.2, K56.600, K56.601, K56.609 |
| cardiotoxicity | I50.814, I50.9, I50.1, I50.810, I50.811, I50.811, I50.812, I50.813, I50.82, I50.83, I50.84, I50.89, I24.8, I50.2X, I50.3X, I20.0, I24.0, I24.8, I49.9, I48.91, I48.92, I48.92, I47.1, I49.5, R00.1, I49.8 |
| Respiratory | J95.859, J95.88, J95.89, J95.89, J98.4, J95.2, J13, J18.1, J18.1, J15.9, J12.9, J17, J18.9, J18.2, J81.1, J15.8 |
| neurological disorders | G62.82, S44.21XS |
| skin disorders | L56.8, L57.8, L57.9, L58.0, L58.1, L58.9, L59.8 |
| lung diseases | J70.0, J70.1 |
| lymphedema | I89.0, I97.2 |
| Impaired Shoulder Joint Mobility | S14.3XXA, S14.3XXD, S14.3XXS, G54.0, G54.5 |
| Unknow | T66.XXXA, T66.XXXD, T66.XXXS, W88.1XXS |
| **Clinical Characteristics** | |
| Metastatic status | C77, C78, C79, C7B, C800 |
| Breast, conserving, surgery ^a^ | 0HBT0ZZ, 0HBT3ZZ, 0HBT7ZZ, 0HBT8ZZ, 0HBTXZZ, 0HBU0ZZ, 0HBU3ZZ, 0HBU7ZZ, 0HBU8ZZ, 0HBUXZZ, 0HBV0ZZ, 0HBV3ZZ, 0HBV7ZZ, 0HBV8ZZ, 0HBVXZZ, 0HBYXZZ |
| Mastectomy ^b^ | 0HTT0ZZ, 0HTU0ZZ, 0HTV0ZZ |
| Chemotherapy ^c^ | 3E00X05, 3E00X0M, 3E01305, 3E0130M, 3E02305, 3E0230M, 3E03002, 3E03003, 3E03005, 3E0300M, 3E0300P, 3E030GN, 3E03302, 3E03303, 3E03305, 3E0330M, 3E0330P, 3E033GN, 3E04002, 3E04003, 3E04005, 3E0400M, 3E0400P, 3E040GN, 3E04302, 3E04303, 3E04305, 3E0430M, 3E0430P, 3E043GN, 3E05002, 3E05003, 3E05005, 3E0500M, 3E0500P, 3E050GN, 3E05302, 3E05303, 3E05305, 3E0530M, 3E0530P, 3E053GN, 3E06002, 3E06003, 3E06005, 3E0600M, 3E0600P, 3E060GN, 3E06302, 3E06303, 3E06305, 3E0630M, 3E0630P, 3E063GN, 3E09305, 3E0930M, 3E09705, 3E0970M, 3E09X05, 3E09X0M, 3E0A305, 3E0A30M, 3E0B305, 3E0B30M, 3E0B705, 3E0B70M, 3E0BX05, 3E0BX0M, 3E0C305, 3E0C30M, 3E0C705, 3E0C70M, 3E0CX05, 3E0CX0M, 3E0D305, 3E0D30M, 3E0D705, 3E0D70M, 3E0DX05, 3E0DX0M, 3E0E305, 3E0E30M, 3E0E705, 3E0E70M, 3E0E805, 3E0E80M, 3E0F305, 3E0F30M, 3E0F705, 3E0F70M, 3E0F805, 3E0F80M, 3E0G305, 3E0G30M, 3E0G705, 3E0G70M, 3E0G805, 3E0G80M, 3E0H305, 3E0H30M, 3E0H705, 3E0H70M, 3E0H805, 3E0H80M, 3E0J305, 3E0J30M, 3E0J705, 3E0J70M, 3E0J805, 3E0J80M, 3E0K305, 3E0K30M, 3E0K705, 3E0K70M, 3E0K805, 3E0K80M, 3E0L305, 3E0L30M, 3E0L705, 3E0L70M, 3E0M305, 3E0M30M, 3E0M705, 3E0M70M, 3E0N305, 3E0N30M, 3E0N705, 3E0N70M, 3E0N805, 3E0N80M, 3E0P305, 3E0P30M, 3E0P705, 3E0P70M, 3E0P805, 3E0P80M, 3E0Q305, 3E0Q30M, 3E0Q705, 3E0Q70M, 3E0R302, 3E0R303, 3E0R305, 3E0R30M, 3E0S302, 3E0S303, 3E0S305, 3E0S30M, 3E0U305, 3E0U30M, 3E0V305, 3E0V30M, 3E0W305, 3E0W30M, 3E0Y305, 3E0Y30M, 3E0Y705, 3E0Y70M, XW03351, XW04351 |

^a^ Procedure, Codes, were, defined, based, on, CCS, procedure, category, (breast, conserving, surgery, [166]), from, https://www.hcup-us.ahrq.gov/toolssoftware/ccsr/prccsr.jsp#download.

https://www.hcup-us.ahrq.gov/toolssoftware/ccs/CCSUsersGuide.pdf

^b^ Procedure, Codes, were, defined, based, on, CCS, procedure, category: mastectomy, (167).

^c^ Procedure, Codes, were, defined, based, on, CCS, procedure, category: cancer, chemotherapy, (224).

**Table S2.** Baseline characteristics of BC patients stratified by radiotherapy and brachytherapy treatment, 2016-2021.

|  | **Overall**  **N=9030** | **Radiotherapy-only**  **N=8297** | **Brachytherapy ± Radiotherapy**  **N=733** | **P-value** |
| --- | --- | --- | --- | --- |
|  | N (%) | N (%) | N (%) |  |
| **Age (y), median (IQR)** | 61 (51, 70) | 61 (51, 71) | 59 (49, 68) | <0.001 |
| **Age at admission** |  |  |  | 0.002 |
| 18~24 | 7 (0.1%) | 5 (0.1%) | 2 (0.3%) |  |
| 25~34 | 215 (2.4%) | 203 (2.4%) | 12 (1.6%) |  |
| 35~44 | 911 (10.1%) | 813 (9.8%) | 98 (13.4%) |  |
| 45~54 | 1784 (19.8%) | 1628 (19.6%) | 156 (21.3%) |  |
| 55~64 | 2416 (26.8%) | 2203 (26.6%) | 213 (29.1%) |  |
| 65~74 | 2217 (24.6%) | 2047 (24.7%) | 170 (23.2%) |  |
| 75+ | 1480 (16.4%) | 1398 (16.8%) | 82 (11.2%) |  |
| **Year** |  |  |  | <0.001 |
| 2016 | 925 (10.2%) | 787 (9.5%) | 138 (18.8%) |  |
| 2017 | 1700 (18.8%) | 1554 (18.7%) | 146 (19.9%) |  |
| 2018 | 1657 (18.3%) | 1514 (18.2%) | 143 (19.5%) |  |
| 2019 | 1771 (19.6%) | 1631 (19.7%) | 140 (19.1%) |  |
| 2020 | 1492 (16.5%) | 1402 (16.9%) | 90 (12.3%) |  |
| 2021 | 1485 (16.4%) | 1409 (17.0%) | 76 (10.4%) |  |
| **LOS, median (IQR)** | 3 (2, 7) | 4 (2, 8) | 2 (1, 3) | <0.001 |
| **Cost, Mean (SD)** | 25380.5 (34422.9) | 25624.5 (35472.5) | 22622.7 (18651.9) | 0.078 |
| **Insurance type** |  |  |  | <0.001 |
| Medicare | 4004 (44.4%) | 3747 (45.2%) | 257 (35.1%) |  |
| Medicaid | 1345 (14.9%) | 1256 (15.2%) | 89 (12.1%) |  |
| Private insurance | 3401 (37.7%) | 3037 (36.6%) | 364 (49.7%) |  |
| Self-pay | 83 (0.9%) | 79 (1.0%) | 4 (0.5%) |  |
| No charge | 11 (0.1%) | 10 (0.1%) | 1 (0.1%) |  |
| Other | 176 (2.0%) | 158 (1.9%) | 18 (2.5%) |  |
| **Income quartile** |  |  |  | 0.044 |
| 0-25th percentile | 1909 (21.4%) | 1785 (21.8%) | 124 (17.1%) |  |
| 26th to 50th percentile | 2107 (23.6%) | 1909 (23.3%) | 198 (27.3%) |  |
| 26th to 50th percentile | 2264 (25.4%) | 2090 (25.5%) | 174 (24.0%) |  |
| 76th to 100th percentile | 2635 (29.6%) | 2407 (29.4%) | 228 (31.5%) |  |
| **CCI** |  |  |  | <0.001 |
| 0~4 | 1856 (22.1%) | 1550 (20.2%) | 306 (41.9%) |  |
| 5~6 | 1417 (16.8%) | 1233 (16.0%) | 184 (25.2%) |  |
| 7~8 | 975 (11.6%) | 888 (11.6%) | 87 (11.9%) |  |
| 9+ | 4165 (49.5%) | 4012 (52.2%) | 153 (21.0%) |  |
| **Comorbidities** |  |  |  |  |
| COPD | 241 (2.7%) | 240 (2.9%) | 1 (0.1%) | <0.001 |
| Depression | 190 (2.1%) | 179 (2.2%) | 11 (1.5%) | 0.494 |
| Diabetes | 970 (10.7%) | 888 (10.7%) | 82 (11.2%) | 0.921 |
| Hypertension | 462 (5.1%) | 443 (5.3%) | 19 (2.6%) | 0.005 |
| Hypothyroidism | 1296 (14.4%) | 1230 (14.8%) | 66 (9.0%) | <0.001 |
| Alcohol abuse | 79 (0.9%) | 78 (0.9%) | 1 (0.1%) | 0.081 |
| Smoker | 2540 (28.1%) | 2355 (28.4%) | 185 (25.2%) | 0.193 |
| Obesity | 864 (9.6%) | 791 (9.5%) | 73 (10.0%) | 0.932 |
| Anemic-deficiency | 1345 (14.9%) | 1304 (15.7%) | 41 (5.6%) | <0.001 |
| Coagulopathy | 352 (3.9%) | 346 (4.2%) | 6 (0.8%) | <0.001 |
| Neutropenia | 384 (4.3%) | 382 (4.6%) | 2 (0.3%) | <0.001 |
| Nausea and Vomiting | 457 (5.1%) | 430 (5.2%) | 27 (3.7%) | 0.207 |
| Metabolic disorders | 130 (1.4%) | 130 (1.6%) | 0 (0.0%) | 0.003 |
| AKI | 839 (9.3%) | 826 (10.0%) | 13 (1.8%) | <0.001 |
| CKD | 360 (4.0%) | 346 (4.2%) | 14 (1.9%) | 0.011 |
| Coronary Artery Disease | 797 (8.8%) | 768 (9.3%) | 29 (4.0%) | <0.001 |
| Atrial Fibrillation | 374 (4.1%) | 365 (4.4%) | 9 (1.2%) | <0.001 |
| Liver Disease | 268 (3.0%) | 257 (3.1%) | 11 (1.5%) | 0.051 |
| Pneumonia | 643 (7.1%) | 643 (7.7%) | 0 (0.0%) | <0.001 |
| Congestive | 877 (9.7%) | 847 (10.2%) | 30 (4.1%) | <0.001 |
| Endocarditis | 14 (0.2%) | 14 (0.2%) | 0 (0.0%) | 0.538 |
| Personal history of irradiation | 1773 (19.6%) | 1715 (20.7%) | 58 (7.9%) | <0.001 |
| **Discharge quarter** |  |  |  | 0.486 |
| Jan.-Mar. | 2183 (24.2%) | 2002 (24.1%) | 181 (24.7%) |  |
| Apr.-Jun. | 2193 (24.3%) | 2027 (24.4%) | 166 (22.6%) |  |
| Jul.-Sep. | 2227 (24.7%) | 2023 (24.4%) | 204 (27.8%) |  |
| Oct.-Dec. | 2427 (26.9%) | 2245 (27.1%) | 182 (24.8%) |  |
| **Hospital bed size** |  |  |  | 0.721 |
| Small | 1230 (13.6%) | 1143 (13.8%) | 87 (11.9%) |  |
| Medium | 2157 (23.9%) | 1978 (23.8%) | 179 (24.4%) |  |
| Large | 5643 (62.5%) | 5176 (62.4%) | 467 (63.7%) |  |
| **Hospital location** |  |  |  | 0.624 |
| Large metropolitan areas | 6265 (69.4%) | 5744 (69.2%) | 521 (71.1%) |  |
| Small metropolitan areas | 2463 (27.3%) | 2275 (27.4%) | 188 (25.6%) |  |
| Micropolitan areas | 231 (2.6%) | 209 (2.5%) | 22 (3.0%) |  |
| Not metropolitan or micropolitan | 71 (0.8%) | 69 (0.8%) | 2 (0.3%) |  |
| **Ownership** |  |  |  | 0.001 |
| Government, nonfederal | 1132 (12.5%) | 1037 (12.5%) | 95 (13.0%) |  |
| Private, non-profit | 7106 (78.7%) | 6501 (78.4%) | 605 (82.5%) |  |
| Private, invest-own | 792 (8.8%) | 759 (9.1%) | 33 (4.5%) |  |

Abbreviations: BC, breast cancer; IQR, interquartile range; SD, standard deviation; CCI, Charlson comorbidity index; COPD, chronic obstructive plumeria disease; LOS, length of stay.

**Table S3** Baseline characteristics of BC patients receiving radiotherapy vs. brachytherapy stratified by hospital teaching status, 2016-2021.

|  | **Teaching Hospital**  **N=****7177** | **Non-Teaching Hospital**  **N=1853** | **P-value** |
| --- | --- | --- | --- |
|  | N (%) | N (%) |  |
| **Treatment type, n (%)** |  |  |  |
| Overall | 7177 (100.0%) | 1853 (100.0%) | 1.000 |
| Radiotherapy-only | 6583 (91.7%) | 1714 (92.5%) | 0.298 |
| Brachytherapy ± Radiotherapy | 594 (8.3%) | 139 (7.5%) | 0.298 |
| **Age (y), median (IQR)** | 61 (51, 70) | 64 (54, 73) | <0.001 |
| **Age at admission** |  |  | <0.001 |
| 18~24 | 7 (0.1%) | 0 (0.0%) |  |
| 25~34 | 187 (2.6%) | 28 (1.5%) |  |
| 35~44 | 785 (10.9%) | 126 (6.8%) |  |
| 45~54 | 1455 (20.3%) | 329 (17.8%) |  |
| 55~64 | 1957 (27.3%) | 459 (24.8%) |  |
| 65~74 | 1694 (23.6%) | 523 (28.2%) |  |
| 75+ | 1092 (15.2%) | 388 (20.9%) |  |
| **Year** |  |  | <0.001 |
| 2016 | 693 (9.7%) | 232 (12.5%) |  |
| 2017 | 1273 (17.7%) | 427 (23.0%) |  |
| 2018 | 1294 (18.0%) | 363 (19.6%) |  |
| 2019 | 1466 (20.4%) | 305 (16.5%) |  |
| 2020 | 1226 (17.1%) | 266 (14.4%) |  |
| 2021 | 1225 (17.1%) | 260 (14.0%) |  |
| **LOS, Mean (SD)** | 4 (2, 8) | 3 (2, 6) | <0.001 |
| **Insurance type** |  |  | <0.001 |
| Medicare | 3027 (42.2%) | 977 (52.8%) |  |
| Medicaid | 1134 (15.8%) | 211 (11.4%) |  |
| Private insurance | 2781 (38.8%) | 620 (33.5%) |  |
| Self-pay | 76 (1.1%) | 7 (0.4%) |  |
| No charge | 8 (0.1%) | 3 (0.2%) |  |
| Other | 142 (2.0%) | 34 (1.8%) |  |
| **Income quartile** |  |  | <0.001 |
| 0-25th percentile | 1484 (20.9%) | 425 (23.3%) |  |
| 26th to 50th percentile | 1601 (22.6%) | 506 (27.7%) |  |
| 26th to 50th percentile | 1827 (25.8%) | 437 (23.9%) |  |
| 76th to 100th percentile | 2178 (30.7%) | 457 (25.0%) |  |
| **Clinical characteristic** |  |  |  |
| Metastatic cancer | 7112 (49.5%) | 1640 (44.3%) | <0.001 |
| Breast conservatory surgery | 822 (5.7%) | 286 (7.7%) | <0.001 |
| Mastectomy | 3506 (24.4%) | 960 (25.9%) | 0.066 |
| Chemotherapy | 366 (2.5%) | 20 (0.5%) | <0.001 |
| **CCI** |  |  | 0.002 |
| 0~4 | 1503 (22.6%) | 353 (20.1%) |  |
| 5~6 | 1075 (16.1%) | 342 (19.5%) |  |
| 7~8 | 759 (11.4%) | 216 (12.3%) |  |
| 9+ | 3321 (49.9%) | 844 (48.1%) |  |
| **Comorbidities** |  |  |  |
| COPD | 181 (2.5%) | 60 (3.2%) | 0.104 |
| Depression | 143 (2.0%) | 47 (2.5%) | 0.173 |
| Diabetes | 777 (10.8%) | 193 (10.4%) | 0.641 |
| Hypertension | 357 (5.0%) | 105 (5.7%) | 0.252 |
| Hypothyroidism | 1004 (14.0%) | 292 (15.8%) | 0.058 |
| Alcohol abuse | 60 (0.8%) | 19 (1.0%) | 0.522 |
| Smoker | 2034 (28.3%) | 506 (27.3%) | 0.394 |
| Obesity | 722 (10.1%) | 142 (7.7%) | 0.002 |
| Anemic-deficiency | 1049 (14.6%) | 296 (16.0%) | 0.154 |
| Coagulopathy | 280 (3.9%) | 72 (3.9%) | 1.000 |
| Neutropenia | 315 (4.4%) | 69 (3.7%) | 0.230 |
| Nausea and Vomiting | 371 (5.2%) | 86 (4.6%) | 0.387 |
| Metabolic disorders | 100 (1.4%) | 30 (1.6%) | 0.537 |
| AKI | 679 (9.5%) | 160 (8.6%) | 0.295 |
| CKD | 287 (4.0%) | 73 (3.9%) | 0.960 |
| Coronary Artery Disease | 629 (8.8%) | 168 (9.1%) | 0.717 |
| Atrial Fibrillation | 287 (4.0%) | 87 (4.7%) | 0.202 |
| Liver Disease | 226 (3.1%) | 42 (2.3%) | 0.055 |
| Pneumonia | 484 (6.7%) | 159 (8.6%) | 0.007 |
| Congestive, CHF | 684 (9.5%) | 193 (10.4%) | 0.270 |
| Endocarditis | 11 (0.2%) | 3 (0.2%) | 1.000 |
| Personal history of irradiation | 1460 (20.3%) | 313 (16.9%) | 0.001 |
| **Discharge quarter** |  |  | 0.546 |
| Jan.-Mar. | 1757 (24.5%) | 426 (23.0%) |  |
| Apr.-Jun. | 1731 (24.1%) | 462 (24.9%) |  |
| Jul.-Sep. | 1772 (24.7%) | 455 (24.6%) |  |
| Oct.-Dec. | 1917 (26.7%) | 510 (27.5%) |  |
| **Hospital bed size** |  |  | 0.038 |
| Small | 1007 (14.0%) | 223 (12.0%) |  |
| Medium | 1726 (24.0%) | 431 (23.3%) |  |
| Large | 4444 (61.9%) | 1199 (64.7%) |  |
| **Hospital location** |  |  | <0.001 |
| Large metropolitan areas | 5370 (74.8%) | 895 (48.3%) |  |
| Small metropolitan areas | 1807 (25.2%) | 656 (35.4%) |  |
| Micropolitan areas | 0 (0.0%) | 231 (12.5%) |  |
| Not metropolitan or micropolitan | 0 (0.0%) | 71 (3.8%) |  |
| **Ownership** |  |  | <0.001 |
| Government, nonfederal | 969 (13.5%) | 163 (8.8%) |  |
| Private, non-profit | 5715 (79.6%) | 1391 (75.1%) |  |
| Private, invest-own | 493 (6.9%) | 299 (16.1%) |  |

Abbreviations: BC, breast cancer; IQR, interquartile range; SD, standard deviation; CCI, Charlson comorbidity index; COPD, chronic obstructive plumeria disease; LOS, length of stay.

**Table S4.** Unadjusted clinical outcomes of in-hospital mortality, readmission and complications stratified by hospital teaching status, 2016-2021.

|  | **Non-Teaching hospital vs. Teaching hospital** | | |
| --- | --- | --- | --- |
| **Outcomes** | **Odds Ratio** | **95%Cl** | **P-value** |
| **Overall** |  |  |  |
| In-hospital mortality | 0.87 | 0.66, 1.14 | 0.316 |
| Radiation-related complication | 1.13 | 1.02, 1.26 | 0.018 |
| Readmission outcomes |  |  |  |
| 30-day readmission | 0.84 | 0.72, 0.97 | 0.016 |
| 90-day readmission | 0.86 | 0.77, 0.98 | 0.020 |
| **Radiotherapy-only** |  |  |  |
| In-hospital mortality | 0.87 | 0.66, 1.14 | 0.310 |
| Radiation-related complication | 1.11 | 1.01, 1.21 | 0.058 |
| Readmission outcomes |  |  |  |
| 30-day readmission | 0.82 | 0.70, 0.95 | 0.008 |
| 90-day readmission | 0.84 | 0.74, 0.95 | 0.005 |
| **Brachytherapy ± Radiotherapy** |  |  |  |
| In-hospital mortality | 0.00 | NA | 0.995 |
| Radiation-related complication | 1.59 | 0.97, 2.62 | 0.068 |
| Readmission outcomes |  |  |  |
| 30-day readmission | 1.26 | 0.58, 2.71 | 0.558 |
| 90-day readmission | 1.55 | 0.88, 2.74 | 0.132 |

Abbreviations: OR, odds ratio; Radiation-related complication, include radiation-induced wounds, infections, strokes, thrombotic events, gastrointestinal issues, cardiotoxicity and respiratory diseases, neurological disorders, skin conditions, pulmonary diseases, lymphedema, shoulder joint disorders, or unspecified conditions, patients with any of these were classified as having a radiation-related complication; Readmission, readmission analyses consider the time between the end of one admission and the start on the next admission.

**Table S5.** Age-adjusted clinical outcomes of in-hospital mortality, readmission and complications stratified by hospital teaching status, 2016-2021.

|  | **Non-Teaching hospital vs. Teaching hospital** | | |
| --- | --- | --- | --- |
| **Outcomes** | **Odds Ratio** | **95%Cl** | **P-value** |
| **Overall** |  |  |  |
| In-hospital mortality | 0.84 | 0.64, 1.11 | 0.223 |
| Radiation-related complication | 1.07 | 0.97, 1.19 | 0.181 |
| Readmission outcomes |  |  |  |
| 30-day readmission | 0.84 | 0.72, 0.97 | 0.019 |
| 90-day readmission | 0.86 | 0.76, 0.97 | 0.018 |
| **Radiotherapy-only** |  |  |  |
| In-hospital mortality | 0.84 | 0.64, 1.11 | 0.230 |
| Radiation-related complication | 1.06 | 0.95, 1.18 | 0.330 |
| Readmission outcomes |  |  |  |
| 30-day readmission | 0.82 | 0.71, 0.96 | 0.011 |
| 90-day readmission | 0.84 | 0.74, 0.95 | 0.006 |
| **Brachytherapy ± Radiotherapy** |  |  |  |
| In-hospital mortality | 0.00 | NA | 0.995 |
| Radiation-related complication | 1.46 | 0.88, 2.43 | 0.138 |
| Readmission outcomes |  |  |  |
| 30-day readmission | 1.24 | 0.57, 2.67 | 0.588 |
| 90-day readmission | 1.49 | 0.84, 2.65 | 0.170 |

Abbreviations: OR, odds ratio; Radiation-related complication, include radiation-induced wounds, infections, strokes, thrombotic events, gastrointestinal issues, cardiotoxicity and respiratory diseases, neurological disorders, skin conditions, pulmonary diseases, lymphedema, shoulder joint disorders, or unspecified conditions, patients with any of these were classified as having a radiation-related complication; Readmission, readmission analyses consider the time between the end of one admission and the start on the next admission.

**Table S6.** Year-adjusted clinical outcomes of in-hospital mortality, readmission and complications stratified by hospital teaching status, 2016-2021.

|  | **Non-Teaching hospital vs. Teaching hospital** | | |
| --- | --- | --- | --- |
| **Outcomes** | **Odds Ratio** | **95%Cl** | **P-value** |
| **Overall** |  |  |  |
| In-hospital mortality | 0.88 | 0.67, 1.17 | 0.379 |
| Radiation-related complication | 1.18 | 1.06, 1.31 | 0.002 |
| Readmission outcomes |  |  |  |
| 30-day readmission | 0.85 | 0.73, 0.98 | 0.027 |
| 90-day readmission | 0.87 | 0.77, 0.99 | 0.028 |
| **Radiotherapy-only** |  |  |  |
| In-hospital mortality | 0.88 | 0.66, 1.16 | 0.354 |
| Radiation-related complication | 1.15 | 1.03, 1.28 | 0.011 |
| Readmission outcomes |  |  |  |
| 30-day readmission | 0.83 | 0.71, 0.96 | 0.012 |
| 90-day readmission | 0.84 | 0.74, 0.95 | 0.007 |
| **Brachytherapy ± Radiotherapy** |  |  |  |
| In-hospital mortality | 0.00 | NA | 0.996 |
| Radiation-related complication | 1.61 | 0.97, 2.65 | 0.063 |
| Readmission outcomes |  |  |  |
| 30-day readmission | 1.27 | 0.59, 2.73 | 0.542 |
| 90-day readmission | 1.55 | 0.88, 2.75 | 0.130 |

Abbreviations: OR, odds ratio; Radiation-related complication, include radiation-induced wounds, infections, strokes, thrombotic events, gastrointestinal issues, cardiotoxicity and respiratory diseases, neurological disorders, skin conditions, pulmonary diseases, lymphedema, shoulder joint disorders, or unspecified conditions, patients with any of these were classified as having a radiation-related complication; Readmission, readmission analyses consider the time between the end of one admission and the start on the next admission.

**Table S7.** Age- and Year-adjusted clinical outcomes of in-hospital mortality, readmission and complications stratified by hospital teaching status, 2016-2021.

|  | **Non-Teaching hospital vs. Teaching hospital** | | |
| --- | --- | --- | --- |
| **Outcomes** | **Odds Ratio** | **95%Cl** | **P-value** |
| **Overall** |  |  |  |
| In-hospital mortality | 0.86 | 0.65, 1.13 | 0.272 |
| Radiation-related complication | 1.12 | 1.01, 1.24 | 0.040 |
| Readmission outcomes |  |  |  |
| 30-day readmission | 0.85 | 0.73, 0.99 | 0.032 |
| 90-day readmission | 0.87 | 0.77, 0.98 | 0.025 |
| **Radiotherapy-only** |  |  |  |
| In-hospital mortality | 0.85 | 0.65, 1.13 | 0.264 |
| Radiation-related complication | 1.09 | 0.98, 1.22 | 0.104 |
| Readmission outcomes |  |  |  |
| 30-day readmission | 0.83 | 0.72, 0.97 | 0.017 |
| 90-day readmission | 0.84 | 0.74, 0.95 | 0.007 |
| **Brachytherapy ± Radiotherapy** |  |  |  |
| In-hospital mortality | 0.00 | NA | 0.996 |
| Radiation-related complication | 1.48 | 0.89, 2.45 | 0.130 |
| Readmission outcomes |  |  |  |
| 30-day readmission | 1.25 | 0.58, 2.70 | 0.572 |
| 90-day readmission | 1.50 | 0.84, 2.66 | 0.167 |

Abbreviations: OR, odds ratio; Radiation-related complication, include radiation-induced wounds, infections, strokes, thrombotic events, gastrointestinal issues, cardiotoxicity and respiratory diseases, neurological disorders, skin conditions, pulmonary diseases, lymphedema, shoulder joint disorders, or unspecified conditions, patients with any of these were classified as having a radiation-related complication; Readmission, readmission analyses consider the time between the end of one admission and the start on the next admission.

**Table S8.** Clinical characteristic-adjusted clinical outcomes of in-hospital mortality, readmission and complications stratified by hospital teaching status, 2016-2021.

|  | **Non-Teaching hospital vs. Teaching hospital** | | |
| --- | --- | --- | --- |
| **Outcomes** | **Odds Ratio** | **95%Cl** | **P-value** |
| **Overall** |  |  |  |
| In-hospital mortality | 0.95 | 0.72, 1.26 | 0.736 |
| Radiation-related complication | 1.20 | 1.07, 1.34 | 0.001 |
| Readmission outcomes |  |  |  |
| 30-day readmission | 0.89 | 0.77, 1.03 | 0.121 |
| 90-day readmission | 0.92 | 0.81, 1.04 | 0.172 |
| **Radiotherapy-only** |  |  |  |
| In-hospital mortality | 0.96 | 0.73, 1.27 | 0.783 |
| Radiation-related complication | 1.18 | 1.05, 1.32 | 0.005 |
| Readmission outcomes |  |  |  |
| 30-day readmission | 0.88 | 0.75, 1.02 | 0.091 |
| 90-day readmission | 0.89 | 0.79, 1.02 | 0.086 |
| **Brachytherapy ± Radiotherapy** |  |  |  |
| In-hospital mortality | 0.00 | NA | 0.998 |
| Radiation-related complication | 1.58 | 0.95, 2.61 | 0.075 |
| Readmission outcomes |  |  |  |
| 30-day readmission | 1.22 | 0.57, 2.64 | 0.607 |
| 90-day readmission | 1.54 | 0.87, 2.73 | 0.140 |

Abbreviations: OR, odds ratio; Radiation-related complication, include radiation-induced wounds, infections, strokes, thrombotic events, gastrointestinal issues, cardiotoxicity and respiratory diseases, neurological disorders, skin conditions, pulmonary diseases, lymphedema, shoulder joint disorders, or unspecified conditions, patients with any of these were classified as having a radiation-related complication; Readmission, readmission analyses consider the time between the end of one admission and the start on the next admission.

**Table S9.** Hospital characteristic-adjusted clinical outcomes of in-hospital mortality, readmission and complications stratified by hospital teaching status, 2016-2021.

|  | **Non-Teaching hospital vs. Teaching hospital** | | |
| --- | --- | --- | --- |
| **Outcomes** | **Odds Ratio** | **95%Cl** | **P-value** |
| **Overall** |  |  |  |
| In-hospital mortality | 0.85 | 0.63, 1.14 | 0.284 |
| Radiation-related complication | 0.92 | 0.87, 0.98 | 0.980 |
| Readmission outcomes |  |  |  |
| 30-day readmission | 0.83 | 0.71, 0.97 | 0.016 |
| 90-day readmission | 0.86 | 0.75, 1.00 | 0.024 |
| **Radiotherapy-only** |  |  |  |
| In-hospital mortality | 0.85 | 0.63, 1.15 | 0.288 |
| Radiation-related complication | 0.99 | 0.88, 1.11 | 0.802 |
| Readmission outcomes |  |  |  |
| 30-day readmission | 0.81 | 0.69, 0.95 | 0.008 |
| 90-day readmission | 0.83 | 0.73, 0.95 | 0.008 |
| **Brachytherapy ± Radiotherapy** |  |  |  |
| In-hospital mortality | 0.00 | NA | 0.996 |
| Radiation-related complication | 1.31 | 0.77, 2.24 | 0.325 |
| Readmission outcomes |  |  |  |
| 30-day readmission | 1.37 | 0.62, 3.03 | 0.438 |
| 90-day readmission | 1.60 | 0.88, 2.90 | 0.123 |

Abbreviations: OR, odds ratio; Radiation-related complication, include radiation-induced wounds, infections, strokes, thrombotic events, gastrointestinal issues, cardiotoxicity and respiratory diseases, neurological disorders, skin conditions, pulmonary diseases, lymphedema, shoulder joint disorders, or unspecified conditions, patients with any of these were classified as having a radiation-related complication; Readmission, readmission analyses consider the time between the end of one admission and the start on the next admission.

**Table S10.** Clinical outcomes adjusted for age, year, baseline characteristics, clinical comorbidities, and hospital characteristics, stratified by hospital teaching status ,2016-2021.

|  | **Non-Teaching hospital vs. Teaching hospital** | | |
| --- | --- | --- | --- |
| **Outcomes** | **Odds Ratio** | **95%Cl** | **P-value** |
| **Overall** |  |  |  |
| In-hospital mortality | 0.77 | 0.57, 1.04 | 0.091 |
| Radiation-related complication | 1.14 | 1.01, 1.28 | 0.039 |
| Readmission outcomes |  |  |  |
| 30-day readmission | 0.87 | 0.74, 1.01 | 0.059 |
| 90-day readmission | 0.88 | 0.78, 1.00 | 0.044 |
| **Radiotherapy-only** |  |  |  |
| In-hospital mortality | 0.78 | 0.58, 1.06 | 0.108 |
| Radiation-related complication | 1.11 | 0.98, 1.26 | 0.115 |
| Readmission outcomes |  |  |  |
| 30-day readmission | 0.85 | 0.73, 0.99 | 0.037 |
| 90-day readmission | 0.85 | 0.75, 0.97 | 0.015 |
| **Brachytherapy ± Radiotherapy** |  |  |  |
| In-hospital mortality | 0.59 | NA | 1.000 |
| Radiation-related complication | 2.00 | 1.06, 3.78 | 0.033 |
| Readmission outcomes |  |  |  |
| 30-day readmission | 1.38 | 0.55, 3.47 | 0.494 |
| 90-day readmission | 1.83 | 0.95, 3.53 | 0.073 |

Abbreviations: OR, odds ratio; Radiation-related complication, include radiation-induced wounds, infections, strokes, thrombotic events, gastrointestinal issues, cardiotoxicity and respiratory diseases, neurological disorders, skin conditions, pulmonary diseases, lymphedema, shoulder joint disorders, or unspecified conditions, patients with any of these were classified as having a radiation-related complication; Readmission, readmission analyses consider the time between the end of one admission and the start on the next admission.

**Table S11.** Adjusted and unadjusted multivariable quantile regression model: median net increase in hospital cost and length of stay.

|  | | **LOS** | | | | | | | |
| --- | --- | --- | --- | --- | --- | --- | --- | --- | --- |
|  | | **Unadjusted** | | **Adjusted** | | | | | |
|  | | **Median (IQR)** | **Median Net Increase**  **In LOS (Days,95%CI)** | **Median Net Increase**  **In LOS (Days,95%CI)**  **Clinical ^a^** | | **Median Net Increase**  **In LOS (Days,95%CI)**  **Hospital ^b^** | | **Median Net Increase**  **In LOS (Days,95%CI)**  **Fully ^c^** | |
| **Overall** | |  |  |  | |  | |  | |
| Teaching hospital | | 4 (2,8) | reference | reference | | reference | | reference | |
| Non-Teaching hospital | | 3 (2,6) | -0.8 (-0.8, -0.8) | -0.4 (-0.7, -0.2) | | -1.6 (-2.2, -0.9) | | -0.5 (-0.6, -0.4) | |
| **Radiotherapy-only** | |  |  |  | |  | |  | |
| Teaching hospital | | 4 (2,8) | reference | reference | | reference | | reference | |
| Non-Teaching hospital | | 3 (2,7) | -0.9 (-1.4, -0.4) | -0.5 (-0.8, -0.1) | | -1.1 (-1.2, -1.0) | | -0.6 (-0.7, -0.4) | |
| **Brachytherapy ± Radiotherapy** | |  |  |  | |  | |  | |
| Teaching hospital | | 2 (1,3) | reference | reference | | reference | | reference | |
| Non-Teaching hospital | | 2 (1,2) | -0.1 (-0.4, 0.3) | -0.1 (-0.6, 0.4) | | -0.2 (-0.3, -0.1) | | -0.0 (-0.3, 0.2) | |
|  | **Cost, in 1,000 US dollars** | | | | | | | | |
|  | **Unadjusted** | | | | **Adjusted** | | | | |
|  | **Median (IQR)** | | **Median Net Increase**  **In cost ($,95%CI)** | | **Median Net Increase**  **In LOS (Days,95%CI)**  **clinical** | | **Median Net Increase**  **In LOS (Days,95%CI)**  **hospital** | | **Median Net Increase**  **In LOS (Days,95%CI)**  **fully** |
| **Overall** |  | |  | |  | |  | |  |
| Teaching hospital | 17.3 (9.2,30.8) | | reference | | reference | | reference | | reference |
| Non-Teaching hospital | 14.5 (7.5,27.0) | | -3.6 (-4.7, -2.6) | | -2.9 (-3.9, -2.0) | | -1.1 (-2.1, 0.1) | | -0.2 (-1.0, 0.6) |
| **Radiotherapy-only** |  | |  | |  | |  | |  |
| Teaching hospital | 17.3 (9.7,31.0) | | reference | | reference | | reference | | reference |
| Non-Teaching hospital | 13.9 (7.3,26.8) | | -4.0 (-5.1, -3.0) | | -3.3 (-4.3, -2.3) | | -1.2 (-2.2, -0.1) | | -0.3 (-1.1, 0.5) |
| **Brachytherapy ± Radiotherapy** |  | |  | |  | |  | |  |
| Teaching hospital | 17.9 (10.3,28.6) | | reference | | reference | | reference | | reference |
| Non-Teaching hospital | 17.6 (10.5,29.7) | | 0.5 (-2.1, 3.0) | | 0.5 (-0.6, 0.4) | | 0.0 (-3.2, 3.3) | | 0.5 (-2.3, 3.2) |

Abbreviations: LOS, length of stay; IQR, interquartile range.

**^a^** Adjusted for clinical characteristics (including Metastatic cancer, breast conservatory surgery, mastectomy and chemotherapy);

^b^ Adjusted for hospital characteristics (including hospital bed size, hospital location and hospital ownership)

^c^ Adjusted for age, year, baseline comorbidities (including COPD, Depression, Diabetes, Hypertension, Hypothyroidism, Alcohol abuse, Smoker, Obesity, Anemic-deficiency, Coagulopathy, Neutropenia, Nausea and Vomiting, Metabolic disorders, AKI, CKD, Coronary Artery Disease, Atrial Fibrillation, Liver Disease, Pneumonia, Congestive, Endocarditis), personal history of irradiation , clinical characteristics (including Metastatic cancer, breast conservatory surgery, mastectomy and chemotherapy) and hospital characteristics (including hospital bed size, hospital location and hospital ownership)

**Table S12.** Akaike information criterion values for models of clinical outcomes by hospital teaching status, 2016-2021.

|  | **Model 1^a^** | **Model 2^b^** | **Model 3^c^** | **Model 4^d^** | **Model 5^e^** | **Model 6^f^** |
| --- | --- | --- | --- | --- | --- | --- |
|  | **AIC** | **AIC** | **AIC** | **AIC** | **AIC** | **AIC** |
| **Overall** | | | | | |  |
| In-hospital mortality | 2951.55 | 2947.23 | 2641.19 | 2852.63 | 2636.56 | 2621.38 |
| Radiation-related complication | 12224.44 | 12032.92 | 10850.22 | 11177.09 | 10684.77 | 10680.72 |
| Readmission outcomes |  |  |  |  |  |  |
| 30-day readmission | 7842.31 | 7839.49 | 7478.88 | 7843.73 | 7469.34 | 7457.24 |
| 90-day readmission | 10009.22 | 10003.83 | 9475.91 | 9751.90 | 9466.66 | 9452.80 |
| **Radiotherapy-only** | | | | | |  |
| In-hospital mortality | 2878.85 | 2877.54 | 2611.69 | 2831.2 | 2609.00 | 2593.48 |
| Radiation-related complication | 11361.26 | 11216.00 | 10249.01 | 11020.44 | 10103.06 | 10098.18 |
| Readmission outcomes |  |  |  |  |  |  |
| 30-day readmission | 7451.4 | 7451.63 | 7162.1 | 7452.37 | 7151.16 | 7138.79 |
| 90-day readmission | 9456.62 | 9429.86 | 8992.99 | 9423.37 | 8984.76 | 8975.89 |
| **Brachytherapy ± Radiotherapy** | | | | | |  |
| In-hospital mortality | 30.77 | 28.87 | 32.14 | 30.44 | 48.00 | 22.00 |
| Radiation-related complication | 573.75 | 560.48 | 550.46 | 543.55 | 552.05 | 532.04 |
| Readmission outcomes |  |  |  |  |  |  |
| 30-day readmission | 314.11 | 316.36 | 313.06 | 318.7 | 328.31 | 302.92 |
| 90-day readmission | 483.76 | 465.74 | 463.17 | 469.21 | 486.69 | 458.83 |

Abbreviations: OR, odds ratio; Radiation-related complication, include radiation-induced wounds, infections, strokes, thrombotic events, gastrointestinal issues, cardiotoxicity and respiratory diseases, neurological disorders, skin conditions, pulmonary diseases, lymphedema, shoulder joint disorders, or unspecified conditions, patients with any of these were classified as having a radiation-related complication; Readmission, readmission analyses consider the time between the end of one admission and the start on the next admission.

**^a^** Unadjusted original logistic regression model;

^b^ Adjusted for both age and year;

^c^ Adjusted for clinical characteristics (including Metastatic cancer, breast conservatory surgery, mastectomy and chemotherapy);

^d^ Adjusted for hospital characteristics (including hospital bed size, hospital location and hospital ownership);

^e^ Fully adjusted for age, year, baseline comorbidities (including COPD, Depression, Diabetes, Hypertension, Hypothyroidism, Alcohol abuse, Smoker, Obesity, Anemic-deficiency, Coagulopathy, Neutropenia, Nausea and Vomiting, Metabolic disorders, AKI, CKD, Coronary Artery Disease, Atrial Fibrillation, Liver Disease, Pneumonia, Congestive, Endocarditis), personal history of irradiation , clinical characteristics (including Metastatic cancer, breast conservatory surgery, mastectomy and chemotherapy) and hospital characteristics (including hospital bed size, hospital location and hospital ownership)

^f^ Model optimized from Model 5 through stepwise regression, with final adjusted variables selected based on the optimal AIC; see Supplementary Table S13 for detailed variable selection.

**Table S13.** Stepwise regression results for Model 6: Selected variables, AIC values, and p-values for clinical outcomes by hospital teaching status and treatment group, 2016-2021.

|  | **Model 6^a^** |  |  |
| --- | --- | --- | --- |
|  | **Variables** | **AIC** | **P-value** |
| **Overall** |  |  |  |
| In-hospital mortality | Hospital bed size, diabetes, hypothyroidism, smoker, obesity, coronary artery disease, metastatic cancer, breast conservatory surgery, mastectomy, personal history of irradiation | 2621.38 | 0.557 |
| Radiation-related complication | Age, year, hospital location, diabetes, hypertension, hypothyroidism, obesity, anemic-deficiency, metastatic cancer, breast conservatory surgery, mastectomy, congestive heart failure, personal history of irradiation | 10680.72 | 0.052 |
| Readmission outcomes |  |  |  |
| 30-day readmission | Age, year, diabetes, hypertension, anemic-deficiency, coronary artery disease, metastatic cancer, breast conservatory surgery, mastectomy | 7457.24 | 0.187 |
| 90-day readmission | Age, hospital ownership, diabetes, hypertension, smoker, anemic-deficiency, nausea and vomiting, coronary artery disease, metastatic cancer, breast conservatory surgery, mastectomy, congestive heart failure | 9452.80 | 0.239 |
| **Radiotherapy-only** |  |  |  |
| In-hospital mortality | Hospital bed size, diabetes, personal history of irradiation, smoker, obesity, hypothyroidism, coronary artery disease, breast conservatory surgery, metastatic cancer, mastectomy | 2593.48 | 0.599 |
| Radiation-related complication | Age, year, hospital location, diabetes, hypertension, hypothyroidism, obesity, anemic-deficiency, nausea and vomiting, metastatic cancer, breast conservatory surgery, mastectomy, congestive heart failure, personal history of irradiation | 10098.18 | 0.090 |
| Readmission outcomes |  |  |  |
| 30-day readmission | Age, year, diabetes, hypertension, anemic-deficiency, coronary artery disease, metastatic cancer, breast conservatory surgery, mastectomy | 7108.79 | 0.141 |
| 90-day readmission | Age, year, diabetes, hypertension, anemic-deficiency, coronary artery disease, metastatic cancer,  breast conservatory surgery, mastectomy | 8975.89 | 0.086 |
| **Brachytherapy ± Radiotherapy** |  |  |  |
| In-hospital mortality | Hospital bed size, diabetes, hypertension, hypothyroidism, smoker, obesity, coronary artery disease, congestive heart failure, personal history of irradiation | 22.00 | 0.996 |
| Radiation-related complication | Age, hospital location, smoker, nausea and vomiting, coronary artery disease, mastectomy, personal history of irradiation | 532.04 | 0.136 |
| Readmission outcomes |  |  |  |
| 30-day readmission | Hospital location, obesity, mastectomy, congestive heart failure | 302.92 | 0.633 |
| 90-day readmission | Smoker, congestive heart failure |  |  |

^a^ Model optimized from Model 5 through stepwise regression, with final adjusted variables selected based on the optimal AIC; see Supplementary Table S13 for detailed variable selection.

**Table S14.** International classification of diseases, tenth edition codes related to radiotherapy.

| **ICD.10.PCS.Code** | **ICD.10.PCS.Code. Description** |
| --- | --- |
| DM000ZZ | DM000ZZ: Beam Radiation of Left Breast using Photons <1 MeV |
| DM001ZZ | DM001ZZ: Beam Radiation of Left Breast using Photons 1 - 10 MeV |
| DM002ZZ | DM002ZZ: Beam Radiation of Left Breast using Photons >10 MeV |
| DM003Z0 | DM003Z0: Beam Radiation of Left Breast using Electrons, Intraop |
| DM003ZZ | DM003ZZ: Beam Radiation of Left Breast using Electrons |
| DM004ZZ | DM004ZZ: Beam Radiation of Left Breast using Heavy Particles |
| DM005ZZ | DM005ZZ: Beam Radiation of Left Breast using Neutrons |
| DM006ZZ | DM006ZZ: Beam Radiation of Left Breast using Neutron Capture |
| DM010ZZ | DM010ZZ: Beam Radiation of Right Breast using Photons <1 MeV |
| DM011ZZ | DM011ZZ: Beam Radiation of Right Breast using Photons 1 - 10 MeV |
| DM012ZZ | DM012ZZ: Beam Radiation of Right Breast using Photons >10 MeV |
| DM013Z0 | DM013Z0: Beam Radiation of Right Breast using Electrons, Intraop |
| DM013ZZ | DM013ZZ: Beam Radiation of Right Breast using Electrons |
| DM014ZZ | DM014ZZ: Beam Radiation of Right Breast using Heavy Particles |
| DM015ZZ | DM015ZZ: Beam Radiation of Right Breast using Neutrons |
| DM016ZZ | DM016ZZ: Beam Radiation of Right Breast using Neutron Capture |
| DM20DZZ | DM20DZZ: Stereotactic Other Photon Radiosurgery of Left Breast |
| DM20HZZ | DM20HZZ: Stereotactic Particulate Radiosurgery of Left Breast |
| DM20JZZ | DM20JZZ: Stereotactic Gamma Beam Radiosurgery of Left Breast |
| DM21DZZ | DM21DZZ: Stereotactic Other Photon Radiosurgery of Right Breast |
| DM21HZZ | DM21HZZ: Stereotactic Particulate Radiosurgery of Right Breast |
| DM21JZZ | DM21JZZ: Stereotactic Gamma Beam Radiosurgery of Right Breast |
| DMY07ZZ | DMY07ZZ: Contact Radiation of Left Breast |
| DMY0FZZ | DMY0FZZ: Plaque Radiation of Left Breast |
| DMY17ZZ | DMY17ZZ: Contact Radiation of Right Breast |
| DMY1FZZ | DMY1FZZ: Plaque Radiation of Right Breast |
| C75LYZZ | C75LYZZ: Nonimag Nucl Med Prob Up Chest Lymph w Oth Radionuclide |
| CW53YZZ | CW53YZZ: Nonimag Nucl Med Prob of Chest using Oth Radionuclide |
| CW54YZZ | CW54YZZ: Nonimag Nucl Med Prob of Chest & Abd using Oth Radionuclide |
| CW56YZZ | CW56YZZ: Nonimag Nucl Med Prob of Chest & Neck using Oth Radionuclide |
| CW73YZZ | CW73YZZ: Sys Nucl Med Therapy of Chest using Oth Radionuclide |
| DB070ZZ | DB070ZZ: Beam Radiation of Chest Wall using Photons <1 MeV |
| DB071ZZ | DB071ZZ: Beam Radiation of Chest Wall using Photons 1 - 10 MeV |
| DB072ZZ | DB072ZZ: Beam Radiation of Chest Wall using Photons >10 MeV |
| DB073Z0 | DB073Z0: Beam Radiation of Chest Wall using Electrons, Intraoperative |
| DB073ZZ | DB073ZZ: Beam Radiation of Chest Wall using Electrons |
| DB074ZZ | DB074ZZ: Beam Radiation of Chest Wall using Heavy Particles |
| DB075ZZ | DB075ZZ: Beam Radiation of Chest Wall using Neutrons |
| DB076ZZ | DB076ZZ: Beam Radiation of Chest Wall using Neutron Capture |
| DB27DZZ | DB27DZZ: Stereotactic Other Photon Radiosurgery of Chest Wall |
| DB27HZZ | DB27HZZ: Stereotactic Particulate Radiosurgery of Chest Wall |
| DB27JZZ | DB27JZZ: Stereotactic Gamma Beam Radiosurgery of Chest Wall |
| DBY77ZZ | DBY77ZZ: Contact Radiation of Chest Wall |
| DBY7FZZ | DBY7FZZ: Plaque Radiation of Chest Wall |
| DH060ZZ | DH060ZZ: Beam Radiation of Chest Skin using Photons <1 MeV |
| DH061ZZ | DH061ZZ: Beam Radiation of Chest Skin using Photons 1 - 10 MeV |
| DH062ZZ | DH062ZZ: Beam Radiation of Chest Skin using Photons >10 MeV |
| DH063Z0 | DH063Z0: Beam Radiation of Chest Skin using Electrons, Intraoperative |
| DH063ZZ | DH063ZZ: Beam Radiation of Chest Skin using Electrons |
| DH064ZZ | DH064ZZ: Beam Radiation of Chest Skin using Heavy Particles |
| DH065ZZ | DH065ZZ: Beam Radiation of Chest Skin using Neutrons |
| DH066ZZ | DH066ZZ: Beam Radiation of Chest Skin using Neutron Capture |
| DHY67ZZ | DHY67ZZ: Contact Radiation of Chest Skin |
| DHY6FZZ | DHY6FZZ: Plaque Radiation of Chest Skin |
| DW020ZZ | DW020ZZ: Beam Radiation of Chest using Photons <1 MeV |
| DW021ZZ | DW021ZZ: Beam Radiation of Chest using Photons 1 - 10 MeV |
| DW022ZZ | DW022ZZ: Beam Radiation of Chest using Photons >10 MeV |
| DW023Z0 | DW023Z0: Beam Radiation of Chest using Electrons, Intraoperative |
| DW023ZZ | DW023ZZ: Beam Radiation of Chest using Electrons |
| DW024ZZ | DW024ZZ: Beam Radiation of Chest using Heavy Particles (Protons,Ions) |
| DW025ZZ | DW025ZZ: Beam Radiation of Chest using Neutrons |
| DW026ZZ | DW026ZZ: Beam Radiation of Chest using Neutron Capture |
| DW22DZZ | DW22DZZ: Stereotactic Other Photon Radiosurgery of Chest |
| DW22HZZ | DW22HZZ: Stereotactic Particulate Radiosurgery of Chest |
| DW22JZZ | DW22JZZ: Stereotactic Gamma Beam Radiosurgery of Chest |
| DWY27ZZ | DWY27ZZ: Contact Radiation of Chest |
| DWY2FZZ | DWY2FZZ: Plaque Radiation of Chest |
| 0WPB41Z | 0WPB41Z: Remove Radioact Elem from L Pleural Cav, Perc Endo |
| 0WPBX1Z | 0WPBX1Z: Removal of Radioact Elem from L Pleural Cav, Extern Approach |
| 0WW901Z | 0WW901Z: Revision of Radioact Elem in R Pleural Cav, Open Approach |
| 0WW931Z | 0WW931Z: Revision of Radioact Elem in R Pleural Cav, Perc Approach |
| 0WW941Z | 0WW941Z: Revise of Radioact Elem in R Pleural Cav, Perc Endo Approach |
| 0WW9X1Z | 0WW9X1Z: Revision of Radioact Elem in R Pleural Cav, Extern Approach |
| 0WWB01Z | 0WWB01Z: Revision of Radioact Elem in L Pleural Cav, Open Approach |
| 0WWB31Z | 0WWB31Z: Revision of Radioact Elem in L Pleural Cav, Perc Approach |
| 0WWB41Z | 0WWB41Z: Revise of Radioact Elem in L Pleural Cav, Perc Endo Approach |
| 0WWBX1Z | 0WWBX1Z: Revision of Radioact Elem in L Pleural Cav, Extern Approach |
| DB050ZZ | DB050ZZ: Beam Radiation of Pleura using Photons <1 MeV |
| DB051ZZ | DB051ZZ: Beam Radiation of Pleura using Photons 1 - 10 MeV |
| DB052ZZ | DB052ZZ: Beam Radiation of Pleura using Photons >10 MeV |
| DB053Z0 | DB053Z0: Beam Radiation of Pleura using Electrons, Intraoperative |
| DB053ZZ | DB053ZZ: Beam Radiation of Pleura using Electrons |
| DB054ZZ | DB054ZZ: Beam Radiation of Pleura using Heavy Particles |
| DB055ZZ | DB055ZZ: Beam Radiation of Pleura using Neutrons |
| DB056ZZ | DB056ZZ: Beam Radiation of Pleura using Neutron Capture |
| DB25DZZ | DB25DZZ: Stereotactic Other Photon Radiosurgery of Pleura |
| DB25HZZ | DB25HZZ: Stereotactic Particulate Radiosurgery of Pleura |
| DB25JZZ | DB25JZZ: Stereotactic Gamma Beam Radiosurgery of Pleura |
| DBY57ZZ | DBY57ZZ: Contact Radiation of Pleura |
| DBY5FZZ | DBY5FZZ: Plaque Radiation of Pleura |
| 0WWC01Z | 0WWC01Z: Revision of Radioact Elem in Mediastinum, Open Approach |
| 0WWC31Z | 0WWC31Z: Revision of Radioact Elem in Mediastinum, Perc Approach |
| 0WWC41Z | 0WWC41Z: Revision of Radioact Elem in Mediastinum, Perc Endo Approach |
| 0WWCX1Z | 0WWCX1Z: Revision of Radioact Elem in Mediastinum, Extern Approach |
| DB060ZZ | DB060ZZ: Beam Radiation of Mediastinum using Photons <1 MeV |
| DB061ZZ | DB061ZZ: Beam Radiation of Mediastinum using Photons 1 - 10 MeV |
| DB062ZZ | DB062ZZ: Beam Radiation of Mediastinum using Photons >10 MeV |
| DB063Z0 | DB063Z0: Beam Radiation of Mediastinum using Electrons, Intraop |
| DB063ZZ | DB063ZZ: Beam Radiation of Mediastinum using Electrons |
| DB064ZZ | DB064ZZ: Beam Radiation of Mediastinum using Heavy Particles |
| DB065ZZ | DB065ZZ: Beam Radiation of Mediastinum using Neutrons |
| DB066ZZ | DB066ZZ: Beam Radiation of Mediastinum using Neutron Capture |
| DB26DZZ | DB26DZZ: Stereotactic Other Photon Radiosurgery of Mediastinum |
| DB26HZZ | DB26HZZ: Stereotactic Particulate Radiosurgery of Mediastinum |
| DB26JZZ | DB26JZZ: Stereotactic Gamma Beam Radiosurgery of Mediastinum |
| DBY67ZZ | DBY67ZZ: Contact Radiation of Mediastinum |
| DBY6FZZ | DBY6FZZ: Plaque Radiation of Mediastinum |
| DB080ZZ | DB080ZZ: Beam Radiation of Diaphragm using Photons <1 MeV |
| DB081ZZ | DB081ZZ: Beam Radiation of Diaphragm using Photons 1 - 10 MeV |
| DB082ZZ | DB082ZZ: Beam Radiation of Diaphragm using Photons >10 MeV |
| DB083Z0 | DB083Z0: Beam Radiation of Diaphragm using Electrons, Intraoperative |
| DB083ZZ | DB083ZZ: Beam Radiation of Diaphragm using Electrons |
| DB084ZZ | DB084ZZ: Beam Radiation of Diaphragm using Heavy Particles |
| DB085ZZ | DB085ZZ: Beam Radiation of Diaphragm using Neutrons |
| DB086ZZ | DB086ZZ: Beam Radiation of Diaphragm using Neutron Capture |
| DB28DZZ | DB28DZZ: Stereotactic Other Photon Radiosurgery of Diaphragm |
| DB28HZZ | DB28HZZ: Stereotactic Particulate Radiosurgery of Diaphragm |
| DB28JZZ | DB28JZZ: Stereotactic Gamma Beam Radiosurgery of Diaphragm |
| DBY87ZZ | DBY87ZZ: Contact Radiation of Diaphragm |
| DBY8FZZ | DBY8FZZ: Plaque Radiation of Diaphragm |
| DW040ZZ | DW040ZZ: Beam Radiation of Hemibody using Photons <1 MeV |
| DW041ZZ | DW041ZZ: Beam Radiation of Hemibody using Photons 1 - 10 MeV |
| DW042ZZ | DW042ZZ: Beam Radiation of Hemibody using Photons >10 MeV |
| DW043Z0 | DW043Z0: Beam Radiation of Hemibody using Electrons, Intraoperative |
| DW043ZZ | DW043ZZ: Beam Radiation of Hemibody using Electrons |
| DW044ZZ | DW044ZZ: Beam Radiation of Hemibody using Heavy Particles |
| DW045ZZ | DW045ZZ: Beam Radiation of Hemibody using Neutrons |
| DW046ZZ | DW046ZZ: Beam Radiation of Hemibody using Neutron Capture |
| DWY47ZZ | DWY47ZZ: Contact Radiation of Hemibody |
| DWY4FZZ | DWY4FZZ: Plaque Radiation of Hemibody |
| CW7NYZZ | CW7NYZZ: Sys Nucl Med Therapy of Whole Body using Oth Radionuclide |
| DW050ZZ | DW050ZZ: Beam Radiation of Whole Body using Photons <1 MeV |
| DW051ZZ | DW051ZZ: Beam Radiation of Whole Body using Photons 1 - 10 MeV |
| DW052ZZ | DW052ZZ: Beam Radiation of Whole Body using Photons >10 MeV |
| DW053Z0 | DW053Z0: Beam Radiation of Whole Body using Electrons, Intraoperative |
| DW053ZZ | DW053ZZ: Beam Radiation of Whole Body using Electrons |
| DW054ZZ | DW054ZZ: Beam Radiation of Whole Body using Heavy Particles |
| DW055ZZ | DW055ZZ: Beam Radiation of Whole Body using Neutrons |
| DW056ZZ | DW056ZZ: Beam Radiation of Whole Body using Neutron Capture |
| DWY57ZZ | DWY57ZZ: Contact Radiation of Whole Body |
| DWY5FZZ | DWY5FZZ: Plaque Radiation of Whole Body |
| DP040ZZ | DP040ZZ: Beam Radiation of Sternum using Photons <1 MeV |
| DP041ZZ | DP041ZZ: Beam Radiation of Sternum using Photons 1 - 10 MeV |
| DP042ZZ | DP042ZZ: Beam Radiation of Sternum using Photons >10 MeV |
| DP043Z0 | DP043Z0: Beam Radiation of Sternum using Electrons, Intraoperative |
| DP043ZZ | DP043ZZ: Beam Radiation of Sternum using Electrons |
| DP044ZZ | DP044ZZ: Beam Radiation of Sternum using Heavy Particles |
| DP045ZZ | DP045ZZ: Beam Radiation of Sternum using Neutrons |
| DP046ZZ | DP046ZZ: Beam Radiation of Sternum using Neutron Capture |
| DPY47ZZ | DPY47ZZ: Contact Radiation of Sternum |
| DPY4FZZ | DPY4FZZ: Plaque Radiation of Sternum |
| D7010ZZ | D7010ZZ: Beam Radiation of Thymus using Photons <1 MeV |
| D7011ZZ | D7011ZZ: Beam Radiation of Thymus using Photons 1 - 10 MeV |
| D7012ZZ | D7012ZZ: Beam Radiation of Thymus using Photons >10 MeV |
| D7013Z0 | D7013Z0: Beam Radiation of Thymus using Electrons, Intraoperative |
| D7013ZZ | D7013ZZ: Beam Radiation of Thymus using Electrons |
| D7014ZZ | D7014ZZ: Beam Radiation of Thymus using Heavy Particles |
| D7015ZZ | D7015ZZ: Beam Radiation of Thymus using Neutrons |
| D7016ZZ | D7016ZZ: Beam Radiation of Thymus using Neutron Capture |
| D721DZZ | D721DZZ: Stereotactic Other Photon Radiosurgery of Thymus |
| D721HZZ | D721HZZ: Stereotactic Particulate Radiosurgery of Thymus |
| D721JZZ | D721JZZ: Stereotactic Gamma Beam Radiosurgery of Thymus |
| D7Y1FZZ | D7Y1FZZ: Plaque Radiation of Thymus |
| D7050ZZ | D7050ZZ: Beam Radiation of Thorax Lymphatics using Photons <1 MeV |
| D7051ZZ | D7051ZZ: Beam Radiation of Thorax Lymphatics using Photons 1 - 10 MeV |
| D7052ZZ | D7052ZZ: Beam Radiation of Thorax Lymphatics using Photons >10 MeV |
| D7053Z0 | D7053Z0: Beam Radiation of Thorax Lymphatics using Electrons, Intraop |
| D7053ZZ | D7053ZZ: Beam Radiation of Thorax Lymphatics using Electrons |
| D7054ZZ | D7054ZZ: Beam Radiation of Thorax Lymphatics using Heavy Particles |
| D7055ZZ | D7055ZZ: Beam Radiation of Thorax Lymphatics using Neutrons |
| D7056ZZ | D7056ZZ: Beam Radiation of Thorax Lymphatics using Neutron Capture |
| D725DZZ | D725DZZ: Stereotactic Other Photon Radiosurgery of Thorax Lymphatics |
| D725HZZ | D725HZZ: Stereotactic Particulate Radiosurgery of Thorax Lymphatics |
| D725JZZ | D725JZZ: Stereotactic Gamma Beam Radiosurgery of Thorax Lymphatics |
| D7Y5FZZ | D7Y5FZZ: Plaque Radiation of Thorax Lymphatics |
| C755YZZ | C755YZZ: Nonimag Nucl Med Prob Head & Neck Lymph w Oth Radionuclide |
| C75DYZZ | C75DYZZ: Nonimag Nucl Med Prob of Pelvic Lymph using Oth Radionuclide |
| C75JYZZ | C75JYZZ: Nonimag Nucl Med Prob of Head Lymph using Oth Radionuclide |
| C75KYZZ | C75KYZZ: Nonimag Nucl Med Prob of Neck Lymph using Oth Radionuclide |
| C75LYZZ | C75LYZZ: Nonimag Nucl Med Prob Up Chest Lymph w Oth Radionuclide |
| C75MYZZ | C75MYZZ: Nonimag Nucl Med Prob of Trunk Lymph using Oth Radionuclide |
| C75NYZZ | C75NYZZ: Nonimag Nucl Med Prob Up Extrem Lymph w Oth Radionuclide |
| C75PYZZ | C75PYZZ: Nonimag Nucl Med Prob Low Extrem Lymph w Oth Radionuclide |
| C75YYZZ | C75YYZZ: Nonimag Nucl Med Prob Lymph & Hemat Sys w Oth Radionuclide |
| C76YYZZ | C76YYZZ: Nonimag Nucl Med Assay Lymph & Hemat Sys w Oth Radionuclide |
| D7030ZZ | D7030ZZ: Beam Radiation of Neck Lymphatics using Photons <1 MeV |
| D7031ZZ | D7031ZZ: Beam Radiation of Neck Lymphatics using Photons 1 - 10 MeV |
| D7032ZZ | D7032ZZ: Beam Radiation of Neck Lymphatics using Photons >10 MeV |
| D7033Z0 | D7033Z0: Beam Radiation of Neck Lymphatics using Electrons, Intraop |
| D7033ZZ | D7033ZZ: Beam Radiation of Neck Lymphatics using Electrons |
| D7034ZZ | D7034ZZ: Beam Radiation of Neck Lymphatics using Heavy Particles |
| D7035ZZ | D7035ZZ: Beam Radiation of Neck Lymphatics using Neutrons |
| D7036ZZ | D7036ZZ: Beam Radiation of Neck Lymphatics using Neutron Capture |
| D7040ZZ | D7040ZZ: Beam Radiation of Axillary Lymphatics using Photons <1 MeV |
| D7041ZZ | D7041ZZ: Beam Radiation of Axilla Lymph using Photons 1 - 10 MeV |
| D7042ZZ | D7042ZZ: Beam Radiation of Axillary Lymphatics using Photons >10 MeV |
| D7043Z0 | D7043Z0: Beam Radiation of Axilla Lymph using Electrons, Intraop |
| D7043ZZ | D7043ZZ: Beam Radiation of Axillary Lymphatics using Electrons |
| D7044ZZ | D7044ZZ: Beam Radiation of Axillary Lymphatics using Heavy Particles |
| D7045ZZ | D7045ZZ: Beam Radiation of Axillary Lymphatics using Neutrons |
| D7046ZZ | D7046ZZ: Beam Radiation of Axillary Lymphatics using Neutron Capture |
| D7050ZZ | D7050ZZ: Beam Radiation of Thorax Lymphatics using Photons <1 MeV |
| D7051ZZ | D7051ZZ: Beam Radiation of Thorax Lymphatics using Photons 1 - 10 MeV |
| D7052ZZ | D7052ZZ: Beam Radiation of Thorax Lymphatics using Photons >10 MeV |
| D7053Z0 | D7053Z0: Beam Radiation of Thorax Lymphatics using Electrons, Intraop |
| D7053ZZ | D7053ZZ: Beam Radiation of Thorax Lymphatics using Electrons |
| D7054ZZ | D7054ZZ: Beam Radiation of Thorax Lymphatics using Heavy Particles |
| D7055ZZ | D7055ZZ: Beam Radiation of Thorax Lymphatics using Neutrons |
| D7056ZZ | D7056ZZ: Beam Radiation of Thorax Lymphatics using Neutron Capture |
| D7060ZZ | D7060ZZ: Beam Radiation of Abdomen Lymphatics using Photons <1 MeV |
| D7061ZZ | D7061ZZ: Beam Radiation of Abd Lymph using Photons 1 - 10 MeV |
| D7062ZZ | D7062ZZ: Beam Radiation of Abdomen Lymphatics using Photons >10 MeV |
| D7063Z0 | D7063Z0: Beam Radiation of Abd Lymph using Electrons, Intraop |
| D7063ZZ | D7063ZZ: Beam Radiation of Abdomen Lymphatics using Electrons |
| D7064ZZ | D7064ZZ: Beam Radiation of Abdomen Lymphatics using Heavy Particles |
| D7065ZZ | D7065ZZ: Beam Radiation of Abdomen Lymphatics using Neutrons |
| D7066ZZ | D7066ZZ: Beam Radiation of Abdomen Lymphatics using Neutron Capture |
| D7070ZZ | D7070ZZ: Beam Radiation of Pelvis Lymphatics using Photons <1 MeV |
| D7071ZZ | D7071ZZ: Beam Radiation of Pelvis Lymphatics using Photons 1 - 10 MeV |
| D7072ZZ | D7072ZZ: Beam Radiation of Pelvis Lymphatics using Photons >10 MeV |
| D7073Z0 | D7073Z0: Beam Radiation of Pelvis Lymphatics using Electrons, Intraop |
| D7073ZZ | D7073ZZ: Beam Radiation of Pelvis Lymphatics using Electrons |
| D7074ZZ | D7074ZZ: Beam Radiation of Pelvis Lymphatics using Heavy Particles |
| D7075ZZ | D7075ZZ: Beam Radiation of Pelvis Lymphatics using Neutrons |
| D7076ZZ | D7076ZZ: Beam Radiation of Pelvis Lymphatics using Neutron Capture |
| D7080ZZ | D7080ZZ: Beam Radiation of Inguinal Lymphatics using Photons <1 MeV |
| D7081ZZ | D7081ZZ: Beam Radiation of Inguinal Lymph using Photons 1 - 10 MeV |
| D7082ZZ | D7082ZZ: Beam Radiation of Inguinal Lymphatics using Photons >10 MeV |
| D7083Z0 | D7083Z0: Beam Radiation of Inguinal Lymph using Electrons, Intraop |
| D7083ZZ | D7083ZZ: Beam Radiation of Inguinal Lymphatics using Electrons |
| D7084ZZ | D7084ZZ: Beam Radiation of Inguinal Lymphatics using Heavy Particles |
| D7085ZZ | D7085ZZ: Beam Radiation of Inguinal Lymphatics using Neutrons |
| D7086ZZ | D7086ZZ: Beam Radiation of Inguinal Lymphatics using Neutron Capture |
| D723DZZ | D723DZZ: Stereotactic Other Photon Radiosurgery of Neck Lymphatics |
| D723HZZ | D723HZZ: Stereotactic Particulate Radiosurgery of Neck Lymphatics |
| D723JZZ | D723JZZ: Stereotactic Gamma Beam Radiosurgery of Neck Lymphatics |
| D724DZZ | D724DZZ: Stereotactic Other Photon Radiosurgery of Axilla Lymph |
| D724HZZ | D724HZZ: Stereotactic Particulate Radiosurgery of Axillary Lymphatics |
| D724JZZ | D724JZZ: Stereotactic Gamma Beam Radiosurgery of Axillary Lymphatics |
| D725DZZ | D725DZZ: Stereotactic Other Photon Radiosurgery of Thorax Lymphatics |
| D725HZZ | D725HZZ: Stereotactic Particulate Radiosurgery of Thorax Lymphatics |
| D725JZZ | D725JZZ: Stereotactic Gamma Beam Radiosurgery of Thorax Lymphatics |
| D726DZZ | D726DZZ: Stereotactic Other Photon Radiosurgery of Abdomen Lymphatics |
| D726HZZ | D726HZZ: Stereotactic Particulate Radiosurgery of Abdomen Lymphatics |
| D726JZZ | D726JZZ: Stereotactic Gamma Beam Radiosurgery of Abdomen Lymphatics |
| D727DZZ | D727DZZ: Stereotactic Other Photon Radiosurgery of Pelvis Lymphatics |
| D727HZZ | D727HZZ: Stereotactic Particulate Radiosurgery of Pelvis Lymphatics |
| D727JZZ | D727JZZ: Stereotactic Gamma Beam Radiosurgery of Pelvis Lymphatics |
| D728DZZ | D728DZZ: Stereotactic Other Photon Radiosurgery of Inguinal Lymph |
| D728HZZ | D728HZZ: Stereotactic Particulate Radiosurgery of Inguinal Lymphatics |
| D728JZZ | D728JZZ: Stereotactic Gamma Beam Radiosurgery of Inguinal Lymphatics |
| D7Y3FZZ | D7Y3FZZ: Plaque Radiation of Neck Lymphatics |
| D7Y4FZZ | D7Y4FZZ: Plaque Radiation of Axillary Lymphatics |
| D7Y5FZZ | D7Y5FZZ: Plaque Radiation of Thorax Lymphatics |
| D7Y6FZZ | D7Y6FZZ: Plaque Radiation of Abdomen Lymphatics |
| D7Y7FZZ | D7Y7FZZ: Plaque Radiation of Pelvis Lymphatics |
| D7Y8FZZ | D7Y8FZZ: Plaque Radiation of Inguinal Lymphatics |

**Table S15.** International classification of diseases, tenth edition codes related to brachytherapy.

| **ICD-10-CM Code** | **ICD-10-CM Code Description** |
| --- | --- |
| Y84.2 | Y84.2: Radiological procedure and radiotherapy as the cause of abnormal reaction of the patient, or of later complication, without mention of misadventure at the time of the procedure |
| Z51.0 | Z51.0: Encounter for antineoplastic radiation therapy |
| W88.1XXA | W88.1XXA: Exposure to radioactive isotopes, initial encounter |
| **ICD.10.PCS.Code** | **ICD.10.PCS.Code. Description** |
| DM1097Z | DM1097Z: HDR Brachytherapy of L Breast using Cesium 137 |
| DM1098Z | DM1098Z: HDR Brachytherapy of L Breast using Iridium 192 |
| DM1099Z | DM1099Z: HDR Brachytherapy of L Breast using Iodine 125 |
| DM109BZ | DM109BZ: High Dose Rate (HDR) Brachytherapy of L Breast using Pd-103 |
| DM109CZ | DM109CZ: HDR Brachytherapy of L Breast using Californium 252 |
| DM109YZ | DM109YZ: HDR Brachytherapy of L Breast using Oth Isotope |
| DM10B6Z | DM10B6Z: LDR Brachytherapy of L Breast using Cesium 131 |
| DM10B7Z | DM10B7Z: LDR Brachytherapy of L Breast using Cesium 137 |
| DM10B8Z | DM10B8Z: LDR Brachytherapy of L Breast using Iridium 192 |
| DM10B9Z | DM10B9Z: LDR Brachytherapy of L Breast using Iodine 125 |
| DM10BB1 | DM10BB1: LDR Brachytherapy of L Breast using Pd-103, Unidirect |
| DM10BBZ | DM10BBZ: Low Dose Rate (LDR) Brachytherapy of L Breast using Pd-103 |
| DM10BCZ | DM10BCZ: LDR Brachytherapy of L Breast using Californium 252 |
| DM10BYZ | DM10BYZ: LDR Brachytherapy of L Breast using Oth Isotope |
| DM1197Z | DM1197Z: HDR Brachytherapy of R Breast using Cesium 137 |
| DM1198Z | DM1198Z: HDR Brachytherapy of R Breast using Iridium 192 |
| DM1199Z | DM1199Z: HDR Brachytherapy of R Breast using Iodine 125 |
| DM119BZ | DM119BZ: High Dose Rate (HDR) Brachytherapy of R Breast using Pd-103 |
| DM119CZ | DM119CZ: HDR Brachytherapy of R Breast using Californium 252 |
| DM119YZ | DM119YZ: HDR Brachytherapy of R Breast using Oth Isotope |
| DM11B6Z | DM11B6Z: LDR Brachytherapy of R Breast using Cesium 131 |
| DM11B7Z | DM11B7Z: LDR Brachytherapy of R Breast using Cesium 137 |
| DM11B8Z | DM11B8Z: LDR Brachytherapy of R Breast using Iridium 192 |
| DM11B9Z | DM11B9Z: LDR Brachytherapy of R Breast using Iodine 125 |
| DM11BB1 | DM11BB1: LDR Brachytherapy of R Breast using Pd-103, Unidirect |
| DM11BBZ | DM11BBZ: Low Dose Rate (LDR) Brachytherapy of R Breast using Pd-103 |
| DM11BCZ | DM11BCZ: LDR Brachytherapy of R Breast using Californium 252 |
| DM11BYZ | DM11BYZ: LDR Brachytherapy of R Breast using Oth Isotope |
| 0HHT01Z | 0HHT01Z: Insertion of Radioact Elem into R Breast, Open Approach |
| 0HHT31Z | 0HHT31Z: Insertion of Radioact Elem into R Breast, Perc Approach |
| 0HHT71Z | 0HHT71Z: Insertion of Radioactive Element into R Breast, Via Opening |
| 0HHT81Z | 0HHT81Z: Insertion of Radioactive Element into Right Breast, Endo |
| 0HHTX1Z | 0HHTX1Z: Insertion of Radioact Elem into R Breast, Extern Approach |
| 0HHU01Z | 0HHU01Z: Insertion of Radioact Elem into L Breast, Open Approach |
| 0HHU31Z | 0HHU31Z: Insertion of Radioact Elem into L Breast, Perc Approach |
| 0HHU71Z | 0HHU71Z: Insertion of Radioactive Element into L Breast, Via Opening |
| 0HHU81Z | 0HHU81Z: Insertion of Radioactive Element into Left Breast, Endo |
| 0HHUX1Z | 0HHUX1Z: Insertion of Radioact Elem into L Breast, Extern Approach |
| 0HHV01Z | 0HHV01Z: Insertion of Radioact Elem into Bi Breast, Open Approach |
| 0HHV31Z | 0HHV31Z: Insertion of Radioact Elem into Bi Breast, Perc Approach |
| 0HHV71Z | 0HHV71Z: Insertion of Radioactive Element into Bi Breast, Via Opening |
| 0HHV81Z | 0HHV81Z: Insertion of Radioactive Element into Bilateral Breast, Endo |
| 0HHVX1Z | 0HHVX1Z: Insertion of Radioact Elem into Bi Breast, Extern Approach |
| 0HPT01Z | 0HPT01Z: Removal of Radioactive Element from R Breast, Open Approach |
| 0HPT31Z | 0HPT31Z: Removal of Radioactive Element from R Breast, Perc Approach |
| 0HPT71Z | 0HPT71Z: Removal of Radioactive Element from R Breast, Via Opening |
| 0HPT81Z | 0HPT81Z: Removal of Radioactive Element from Right Breast, Endo |
| 0HPTX1Z | 0HPTX1Z: Removal of Radioact Elem from R Breast, Extern Approach |
| 0HPU01Z | 0HPU01Z: Removal of Radioactive Element from L Breast, Open Approach |
| 0HPU31Z | 0HPU31Z: Removal of Radioactive Element from L Breast, Perc Approach |
| 0HPU71Z | 0HPU71Z: Removal of Radioactive Element from Left Breast, Via Opening |
| 0HPU81Z | 0HPU81Z: Removal of Radioactive Element from Left Breast, Endo |
| 0HPUX1Z | 0HPUX1Z: Removal of Radioact Elem from L Breast, Extern Approach |
| DB1797Z | DB1797Z: HDR Brachytherapy of Chest Wall using Cesium 137 |
| DB1798Z | DB1798Z: HDR Brachytherapy of Chest Wall using Iridium 192 |
| DB1799Z | DB1799Z: HDR Brachytherapy of Chest Wall using Iodine 125 |
| DB179BZ | DB179BZ: HDR Brachytherapy of Chest Wall using Pd-103 |
| DB179CZ | DB179CZ: HDR Brachytherapy of Chest Wall using Californium 252 |
| DB179YZ | DB179YZ: HDR Brachytherapy of Chest Wall using Oth Isotope |
| DB17B6Z | DB17B6Z: LDR Brachytherapy of Chest Wall using Cesium 131 |
| DB17B7Z | DB17B7Z: LDR Brachytherapy of Chest Wall using Cesium 137 |
| DB17B8Z | DB17B8Z: LDR Brachytherapy of Chest Wall using Iridium 192 |
| DB17B9Z | DB17B9Z: LDR Brachytherapy of Chest Wall using Iodine 125 |
| DB17BB1 | DB17BB1: LDR Brachytherapy of Chest Wall using Pd-103, Unidirect |
| DB17BBZ | DB17BBZ: Low Dose Rate (LDR) Brachytherapy of Chest Wall using Pd-103 |
| DB17BCZ | DB17BCZ: LDR Brachytherapy of Chest Wall using Californium 252 |
| DB17BYZ | DB17BYZ: LDR Brachytherapy of Chest Wall using Oth Isotope |
| DW1297Z | DW1297Z: High Dose Rate (HDR) Brachytherapy of Chest using Cesium 137 |
| DW1298Z | DW1298Z: HDR Brachytherapy of Chest using Iridium 192 |
| DW1299Z | DW1299Z: High Dose Rate (HDR) Brachytherapy of Chest using Iodine 125 |
| DW129BZ | DW129BZ: High Dose Rate (HDR) Brachytherapy of Chest using Pd-103 |
| DW129CZ | DW129CZ: HDR Brachytherapy of Chest using Californium 252 |
| DW129YZ | DW129YZ: HDR Brachytherapy of Chest using Oth Isotope |
| DW12B6Z | DW12B6Z: Low Dose Rate (LDR) Brachytherapy of Chest using Cesium 131 |
| DW12B7Z | DW12B7Z: Low Dose Rate (LDR) Brachytherapy of Chest using Cesium 137 |
| DW12B8Z | DW12B8Z: Low Dose Rate (LDR) Brachytherapy of Chest using Iridium 192 |
| DW12B9Z | DW12B9Z: Low Dose Rate (LDR) Brachytherapy of Chest using Iodine 125 |
| DW12BB1 | DW12BB1: LDR Brachytherapy of Chest using Pd-103, Unidirect |
| DW12BBZ | DW12BBZ: Low Dose Rate (LDR) Brachytherapy of Chest using Pd-103 |
| DW12BCZ | DW12BCZ: LDR Brachytherapy of Chest using Californium 252 |
| DW12BYZ | DW12BYZ: Low Dose Rate (LDR) Brachytherapy of Chest using Oth Isotope |
| 0WH801Z | 0WH801Z: Insertion of Radioact Elem into Chest Wall, Open Approach |
| 0WH831Z | 0WH831Z: Insertion of Radioact Elem into Chest Wall, Perc Approach |
| 0WH841Z | 0WH841Z: Insert of Radioact Elem into Chest Wall, Perc Endo Approach |
| CW73NZZ | CW73NZZ: Sys Nucl Med Therapy of Chest using Phosphorus 32 |
| CW73YZZ | CW73YZZ: Sys Nucl Med Therapy of Chest using Oth Radionuclide |
| DB1597Z | DB1597Z: HDR Brachytherapy of Pleura using Cesium 137 |
| DB1598Z | DB1598Z: HDR Brachytherapy of Pleura using Iridium 192 |
| DB1599Z | DB1599Z: HDR Brachytherapy of Pleura using Iodine 125 |
| DB159BZ | DB159BZ: High Dose Rate (HDR) Brachytherapy of Pleura using Pd-103 |
| DB159CZ | DB159CZ: HDR Brachytherapy of Pleura using Californium 252 |
| DB159YZ | DB159YZ: HDR Brachytherapy of Pleura using Oth Isotope |
| DB15B6Z | DB15B6Z: Low Dose Rate (LDR) Brachytherapy of Pleura using Cesium 131 |
| DB15B7Z | DB15B7Z: Low Dose Rate (LDR) Brachytherapy of Pleura using Cesium 137 |
| DB15B8Z | DB15B8Z: LDR Brachytherapy of Pleura using Iridium 192 |
| DB15B9Z | DB15B9Z: Low Dose Rate (LDR) Brachytherapy of Pleura using Iodine 125 |
| DB15BB1 | DB15BB1: LDR Brachytherapy of Pleura using Pd-103, Unidirect |
| DB15BBZ | DB15BBZ: Low Dose Rate (LDR) Brachytherapy of Pleura using Pd-103 |
| DB15BCZ | DB15BCZ: LDR Brachytherapy of Pleura using Californium 252 |
| DB15BYZ | DB15BYZ: LDR Brachytherapy of Pleura using Oth Isotope |
| 0WH901Z | 0WH901Z: Insertion of Radioact Elem into R Pleural Cav, Open Approach |
| 0WH931Z | 0WH931Z: Insertion of Radioact Elem into R Pleural Cav, Perc Approach |
| 0WHB01Z | 0WHB01Z: Insertion of Radioact Elem into L Pleural Cav, Open Approach |
| 0WHB31Z | 0WHB31Z: Insertion of Radioact Elem into L Pleural Cav, Perc Approach |
| 0WH941Z | 0WH941Z: Insert Radioact Elem in R Pleural Cav, Perc Endo |
| 0WHB41Z | 0WHB41Z: Insert Radioact Elem in L Pleural Cav, Perc Endo |
| 3E0L3HZ | 3E0L3HZ: Introduce of Radioact Subst into Pleural Cav, Perc Approach |
| DB1697Z | DB1697Z: HDR Brachytherapy of Mediastinum using Cesium 137 |
| DB1698Z | DB1698Z: HDR Brachytherapy of Mediastinum using Iridium 192 |
| DB1699Z | DB1699Z: HDR Brachytherapy of Mediastinum using Iodine 125 |
| DB169BZ | DB169BZ: HDR Brachytherapy of Mediastinum using Pd-103 |
| DB169CZ | DB169CZ: HDR Brachytherapy of Mediastinum using Californium 252 |
| DB169YZ | DB169YZ: HDR Brachytherapy of Mediastinum using Oth Isotope |
| DB16B6Z | DB16B6Z: LDR Brachytherapy of Mediastinum using Cesium 131 |
| DB16B7Z | DB16B7Z: LDR Brachytherapy of Mediastinum using Cesium 137 |
| DB16B8Z | DB16B8Z: LDR Brachytherapy of Mediastinum using Iridium 192 |
| DB16B9Z | DB16B9Z: LDR Brachytherapy of Mediastinum using Iodine 125 |
| DB16BB1 | DB16BB1: LDR Brachytherapy of Mediastinum using Pd-103, Unidirect |
| DB16BBZ | DB16BBZ: LDR Brachytherapy of Mediastinum using Pd-103 |
| DB16BCZ | DB16BCZ: LDR Brachytherapy of Mediastinum using Californium 252 |
| DB16BYZ | DB16BYZ: LDR Brachytherapy of Mediastinum using Oth Isotope |
| 0WHC01Z | 0WHC01Z: Insertion of Radioact Elem into Mediastinum, Open Approach |
| 0WHC31Z | 0WHC31Z: Insertion of Radioact Elem into Mediastinum, Perc Approach |
| 0WHC41Z | 0WHC41Z: Insert of Radioact Elem into Mediastinum, Perc Endo Approach |
| DB1897Z | DB1897Z: HDR Brachytherapy of Diaphragm using Cesium 137 |
| DB1898Z | DB1898Z: HDR Brachytherapy of Diaphragm using Iridium 192 |
| DB1899Z | DB1899Z: HDR Brachytherapy of Diaphragm using Iodine 125 |
| DB189BZ | DB189BZ: High Dose Rate (HDR) Brachytherapy of Diaphragm using Pd-103 |
| DB189CZ | DB189CZ: HDR Brachytherapy of Diaphragm using Californium 252 |
| DB189YZ | DB189YZ: HDR Brachytherapy of Diaphragm using Oth Isotope |
| DB18B6Z | DB18B6Z: LDR Brachytherapy of Diaphragm using Cesium 131 |
| DB18B7Z | DB18B7Z: LDR Brachytherapy of Diaphragm using Cesium 137 |
| DB18B8Z | DB18B8Z: LDR Brachytherapy of Diaphragm using Iridium 192 |
| DB18B9Z | DB18B9Z: LDR Brachytherapy of Diaphragm using Iodine 125 |
| DB18BB1 | DB18BB1: LDR Brachytherapy of Diaphragm using Pd-103, Unidirect |
| DB18BBZ | DB18BBZ: Low Dose Rate (LDR) Brachytherapy of Diaphragm using Pd-103 |
| DB18BCZ | DB18BCZ: LDR Brachytherapy of Diaphragm using Californium 252 |
| DB18BYZ | DB18BYZ: LDR Brachytherapy of Diaphragm using Oth Isotope |
| CW7N8ZZ | CW7N8ZZ: Sys Nucl Med Therapy of Whole Body using Samarium 153 |
| CW7NGZZ | CW7NGZZ: Sys Nucl Med Therapy of Whole Body using Iodine 131 |
| CW7NNZZ | CW7NNZZ: Sys Nucl Med Therapy of Whole Body using Phosphorus 32 |
| CW7NPZZ | CW7NPZZ: Sys Nucl Med Therapy of Whole Body using Strontium 89 |
| CW7NYZZ | CW7NYZZ: Sys Nucl Med Therapy of Whole Body using Oth Radionuclide |
| DWY5GDZ | DWY5GDZ: Isotope Administration to Whole Body using Iodine 131 |
| DWY5GFZ | DWY5GFZ: Isotope Administration to Whole Body using Phosphorus 32 |
| DWY5GGZ | DWY5GGZ: Isotope Administration to Whole Body using Strontium 89 |
| DWY5GHZ | DWY5GHZ: Isotope Administration to Whole Body using Strontium 90 |
| DWY5GYZ | DWY5GYZ: Isotope Administration to Whole Body using Other Isotope |
| D71197Z | D71197Z: HDR Brachytherapy of Thymus using Cesium 137 |
| D71198Z | D71198Z: HDR Brachytherapy of Thymus using Iridium 192 |
| D71199Z | D71199Z: HDR Brachytherapy of Thymus using Iodine 125 |
| D7119BZ | D7119BZ: High Dose Rate (HDR) Brachytherapy of Thymus using Pd-103 |
| D7119CZ | D7119CZ: HDR Brachytherapy of Thymus using Californium 252 |
| D7119YZ | D7119YZ: HDR Brachytherapy of Thymus using Oth Isotope |
| D711B6Z | D711B6Z: Low Dose Rate (LDR) Brachytherapy of Thymus using Cesium 131 |
| D711B7Z | D711B7Z: Low Dose Rate (LDR) Brachytherapy of Thymus using Cesium 137 |
| D711B8Z | D711B8Z: LDR Brachytherapy of Thymus using Iridium 192 |
| D711B9Z | D711B9Z: Low Dose Rate (LDR) Brachytherapy of Thymus using Iodine 125 |
| D711BB1 | D711BB1: LDR Brachytherapy of Thymus using Pd-103, Unidirect |
| D711BBZ | D711BBZ: Low Dose Rate (LDR) Brachytherapy of Thymus using Pd-103 |
| D711BCZ | D711BCZ: LDR Brachytherapy of Thymus using Californium 252 |
| D711BYZ | D711BYZ: LDR Brachytherapy of Thymus using Oth Isotope |
| 07HM01Z | 07HM01Z: Insertion of Radioactive Element into Thymus, Open Approach |
| 07HM31Z | 07HM31Z: Insertion of Radioactive Element into Thymus, Perc Approach |
| 07HM41Z | 07HM41Z: Insertion of Radioact Elem into Thymus, Perc Endo Approach |
| D71597Z | D71597Z: HDR Brachytherapy of Thorax Lymph using Cesium 137 |
| D71598Z | D71598Z: HDR Brachytherapy of Thorax Lymph using Iridium 192 |
| D71599Z | D71599Z: HDR Brachytherapy of Thorax Lymph using Iodine 125 |
| D7159BZ | D7159BZ: HDR Brachytherapy of Thorax Lymph using Pd-103 |
| D7159CZ | D7159CZ: HDR Brachytherapy of Thorax Lymph using Californium 252 |
| D7159YZ | D7159YZ: HDR Brachytherapy of Thorax Lymph using Oth Isotope |
| D715B6Z | D715B6Z: LDR Brachytherapy of Thorax Lymph using Cesium 131 |
| D715B7Z | D715B7Z: LDR Brachytherapy of Thorax Lymph using Cesium 137 |
| D715B8Z | D715B8Z: LDR Brachytherapy of Thorax Lymph using Iridium 192 |
| D715B9Z | D715B9Z: LDR Brachytherapy of Thorax Lymph using Iodine 125 |
| D715BB1 | D715BB1: LDR Brachytherapy of Thorax Lymph using Pd-103, Unidirect |
| D715BBZ | D715BBZ: LDR Brachytherapy of Thorax Lymph using Pd-103 |
| D715BCZ | D715BCZ: LDR Brachytherapy of Thorax Lymph using Californium 252 |
| D715BYZ | D715BYZ: LDR Brachytherapy of Thorax Lymph using Oth Isotope |
| D71397Z | D71397Z: HDR Brachytherapy of Neck Lymph using Cesium 137 |
| D71398Z | D71398Z: HDR Brachytherapy of Neck Lymph using Iridium 192 |
| D71399Z | D71399Z: HDR Brachytherapy of Neck Lymph using Iodine 125 |
| D7139BZ | D7139BZ: HDR Brachytherapy of Neck Lymph using Pd-103 |
| D7139CZ | D7139CZ: HDR Brachytherapy of Neck Lymph using Californium 252 |
| D7139YZ | D7139YZ: HDR Brachytherapy of Neck Lymph using Oth Isotope |
| D713B6Z | D713B6Z: LDR Brachytherapy of Neck Lymph using Cesium 131 |
| D713B7Z | D713B7Z: LDR Brachytherapy of Neck Lymph using Cesium 137 |
| D713B8Z | D713B8Z: LDR Brachytherapy of Neck Lymph using Iridium 192 |
| D713B9Z | D713B9Z: LDR Brachytherapy of Neck Lymph using Iodine 125 |
| D713BB1 | D713BB1: LDR Brachytherapy of Neck Lymph using Pd-103, Unidirect |
| D713BBZ | D713BBZ: Low Dose Rate (LDR) Brachytherapy of Neck Lymph using Pd-103 |
| D713BCZ | D713BCZ: LDR Brachytherapy of Neck Lymph using Californium 252 |
| D713BYZ | D713BYZ: LDR Brachytherapy of Neck Lymph using Oth Isotope |
| D71497Z | D71497Z: HDR Brachytherapy of Axilla Lymph using Cesium 137 |
| D71498Z | D71498Z: HDR Brachytherapy of Axilla Lymph using Iridium 192 |
| D71499Z | D71499Z: HDR Brachytherapy of Axilla Lymph using Iodine 125 |
| D7149BZ | D7149BZ: HDR Brachytherapy of Axilla Lymph using Pd-103 |
| D7149CZ | D7149CZ: HDR Brachytherapy of Axilla Lymph using Californium 252 |
| D7149YZ | D7149YZ: HDR Brachytherapy of Axilla Lymph using Oth Isotope |
| D714B6Z | D714B6Z: LDR Brachytherapy of Axilla Lymph using Cesium 131 |
| D714B7Z | D714B7Z: LDR Brachytherapy of Axilla Lymph using Cesium 137 |
| D714B8Z | D714B8Z: LDR Brachytherapy of Axilla Lymph using Iridium 192 |
| D714B9Z | D714B9Z: LDR Brachytherapy of Axilla Lymph using Iodine 125 |
| D714BB1 | D714BB1: LDR Brachytherapy of Axilla Lymph using Pd-103, Unidirect |
| D714BBZ | D714BBZ: LDR Brachytherapy of Axilla Lymph using Pd-103 |
| D714BCZ | D714BCZ: LDR Brachytherapy of Axilla Lymph using Californium 252 |
| D714BYZ | D714BYZ: LDR Brachytherapy of Axilla Lymph using Oth Isotope |
| D71597Z | D71597Z: HDR Brachytherapy of Thorax Lymph using Cesium 137 |
| D71598Z | D71598Z: HDR Brachytherapy of Thorax Lymph using Iridium 192 |
| D71599Z | D71599Z: HDR Brachytherapy of Thorax Lymph using Iodine 125 |
| D7159BZ | D7159BZ: HDR Brachytherapy of Thorax Lymph using Pd-103 |
| D7159CZ | D7159CZ: HDR Brachytherapy of Thorax Lymph using Californium 252 |
| D7159YZ | D7159YZ: HDR Brachytherapy of Thorax Lymph using Oth Isotope |
| D715B6Z | D715B6Z: LDR Brachytherapy of Thorax Lymph using Cesium 131 |
| D715B7Z | D715B7Z: LDR Brachytherapy of Thorax Lymph using Cesium 137 |
| D715B8Z | D715B8Z: LDR Brachytherapy of Thorax Lymph using Iridium 192 |
| D715B9Z | D715B9Z: LDR Brachytherapy of Thorax Lymph using Iodine 125 |
| D715BB1 | D715BB1: LDR Brachytherapy of Thorax Lymph using Pd-103, Unidirect |
| D715BBZ | D715BBZ: LDR Brachytherapy of Thorax Lymph using Pd-103 |
| D715BCZ | D715BCZ: LDR Brachytherapy of Thorax Lymph using Californium 252 |
| D715BYZ | D715BYZ: LDR Brachytherapy of Thorax Lymph using Oth Isotope |
| D71697Z | D71697Z: HDR Brachytherapy of Abd Lymph using Cesium 137 |
| D71698Z | D71698Z: HDR Brachytherapy of Abd Lymph using Iridium 192 |
| D71699Z | D71699Z: HDR Brachytherapy of Abd Lymph using Iodine 125 |
| D7169BZ | D7169BZ: High Dose Rate (HDR) Brachytherapy of Abd Lymph using Pd-103 |
| D7169CZ | D7169CZ: HDR Brachytherapy of Abd Lymph using Californium 252 |
| D7169YZ | D7169YZ: HDR Brachytherapy of Abd Lymph using Oth Isotope |
| D716B6Z | D716B6Z: LDR Brachytherapy of Abd Lymph using Cesium 131 |
| D716B7Z | D716B7Z: LDR Brachytherapy of Abd Lymph using Cesium 137 |
| D716B8Z | D716B8Z: LDR Brachytherapy of Abd Lymph using Iridium 192 |
| D716B9Z | D716B9Z: LDR Brachytherapy of Abd Lymph using Iodine 125 |
| D716BB1 | D716BB1: LDR Brachytherapy of Abd Lymph using Pd-103, Unidirect |
| D716BBZ | D716BBZ: Low Dose Rate (LDR) Brachytherapy of Abd Lymph using Pd-103 |
| D716BCZ | D716BCZ: LDR Brachytherapy of Abd Lymph using Californium 252 |
| D716BYZ | D716BYZ: LDR Brachytherapy of Abd Lymph using Oth Isotope |
| D71797Z | D71797Z: HDR Brachytherapy of Pelvis Lymph using Cesium 137 |
| D71798Z | D71798Z: HDR Brachytherapy of Pelvis Lymph using Iridium 192 |
| D71799Z | D71799Z: HDR Brachytherapy of Pelvis Lymph using Iodine 125 |
| D7179BZ | D7179BZ: HDR Brachytherapy of Pelvis Lymph using Pd-103 |
| D7179CZ | D7179CZ: HDR Brachytherapy of Pelvis Lymph using Californium 252 |
| D7179YZ | D7179YZ: HDR Brachytherapy of Pelvis Lymph using Oth Isotope |
| D717B6Z | D717B6Z: LDR Brachytherapy of Pelvis Lymph using Cesium 131 |
| D717B7Z | D717B7Z: LDR Brachytherapy of Pelvis Lymph using Cesium 137 |
| D717B8Z | D717B8Z: LDR Brachytherapy of Pelvis Lymph using Iridium 192 |
| D717B9Z | D717B9Z: LDR Brachytherapy of Pelvis Lymph using Iodine 125 |
| D717BB1 | D717BB1: LDR Brachytherapy of Pelvis Lymph using Pd-103, Unidirect |
| D717BBZ | D717BBZ: LDR Brachytherapy of Pelvis Lymph using Pd-103 |
| D717BCZ | D717BCZ: LDR Brachytherapy of Pelvis Lymph using Californium 252 |
| D717BYZ | D717BYZ: LDR Brachytherapy of Pelvis Lymph using Oth Isotope |
| D71897Z | D71897Z: HDR Brachytherapy of Inguinal Lymph using Cesium 137 |
| D71898Z | D71898Z: HDR Brachytherapy of Inguinal Lymph using Iridium 192 |
| D71899Z | D71899Z: HDR Brachytherapy of Inguinal Lymph using Iodine 125 |
| D7189BZ | D7189BZ: HDR Brachytherapy of Inguinal Lymph using Pd-103 |
| D7189CZ | D7189CZ: HDR Brachytherapy of Inguinal Lymph using Californium 252 |
| D7189YZ | D7189YZ: HDR Brachytherapy of Inguinal Lymph using Oth Isotope |
| D718B6Z | D718B6Z: LDR Brachytherapy of Inguinal Lymph using Cesium 131 |
| D718B7Z | D718B7Z: LDR Brachytherapy of Inguinal Lymph using Cesium 137 |
| D718B8Z | D718B8Z: LDR Brachytherapy of Inguinal Lymph using Iridium 192 |
| D718B9Z | D718B9Z: LDR Brachytherapy of Inguinal Lymph using Iodine 125 |
| D718BB1 | D718BB1: LDR Brachytherapy of Inguinal Lymph using Pd-103, Unidirect |
| D718BBZ | D718BBZ: LDR Brachytherapy of Inguinal Lymph using Pd-103 |
| D718BCZ | D718BCZ: LDR Brachytherapy of Inguinal Lymph using Californium 252 |
| D718BYZ | D718BYZ: LDR Brachytherapy of Inguinal Lymph using Oth Isotope |
| 07HN01Z | 07HN01Z: Insertion of Radioactive Element into Lymph, Open Approach |
| 07HN31Z | 07HN31Z: Insertion of Radioactive Element into Lymph, Perc Approach |
| 07HN41Z | 07HN41Z: Insertion of Radioact Elem into Lymph, Perc Endo Approach |
| 3E0W3HZ | 3E0W3HZ: Introduction of Radioact Subst into Lymph, Perc Approach |
